# Internal transcription termination widely regulates differential expression of operon-organized genes including ribosomal protein and RNA polymerase genes in an archaeon

**DOI:** 10.1093/nar/gkad575

**Published:** 2023-07-13

**Authors:** Wenting Zhang, Derong Ren, Zhihua Li, Lei Yue, William B Whitman, Xiuzhu Dong, Jie Li

**Affiliations:** State Key Laboratory of Microbial Resources, Institute of Microbiology, Chinese Academy of Sciences, Beijing 100101, PR China; University of Chinese Academy of Sciences, No.19A Yuquan Road, Shijingshan District, Beijing 100049, China; State Key Laboratory of Microbial Resources, Institute of Microbiology, Chinese Academy of Sciences, Beijing 100101, PR China; University of Chinese Academy of Sciences, No.19A Yuquan Road, Shijingshan District, Beijing 100049, China; State Key Laboratory of Microbial Resources, Institute of Microbiology, Chinese Academy of Sciences, Beijing 100101, PR China; University of Chinese Academy of Sciences, No.19A Yuquan Road, Shijingshan District, Beijing 100049, China; State Key Laboratory of Microbial Resources, Institute of Microbiology, Chinese Academy of Sciences, Beijing 100101, PR China; University of Chinese Academy of Sciences, No.19A Yuquan Road, Shijingshan District, Beijing 100049, China; Department of Microbiology, University of Georgia, Athens, GA, USA; State Key Laboratory of Microbial Resources, Institute of Microbiology, Chinese Academy of Sciences, Beijing 100101, PR China; University of Chinese Academy of Sciences, No.19A Yuquan Road, Shijingshan District, Beijing 100049, China; State Key Laboratory of Microbial Resources, Institute of Microbiology, Chinese Academy of Sciences, Beijing 100101, PR China

## Abstract

Genes organized within operons in prokaryotes benefit from coordinated expression. However, within many operons, genes are expressed at different levels, and the mechanisms for this remain obscure. By integrating PacBio-seq, dRNA-seq, Term-seq and Illumina-seq data of a representative archaeon *Methanococcus maripaludis*, internal transcription termination sites (ioTTSs) were identified within 38% of operons. Higher transcript and protein abundances were found for genes upstream than downstream of ioTTSs. For representative operons, these differences were confirmed by northern blotting, qRT-PCR and western blotting, demonstrating that these ioTTS terminations were functional. Of special interest, mutation of ioTTSs in ribosomal protein (RP)-RNA polymerase (RNAP) operons not only elevated expression of the downstream RNAP genes but also decreased production of the assembled RNAP complex, slowed whole cell transcription and translation, and inhibited growth. Overexpression of the RNAP subunits with a shuttle vector generated the similar physiological effects. Therefore, ioTTS termination is a general and physiologically significant regulatory mechanism of the operon gene expression. Because the RP-RNAP operons are found to be widely distributed in archaeal species, this regulatory mechanism could be commonly employed in archaea.

## Graphical Abstract

**Graphical Abstract:**
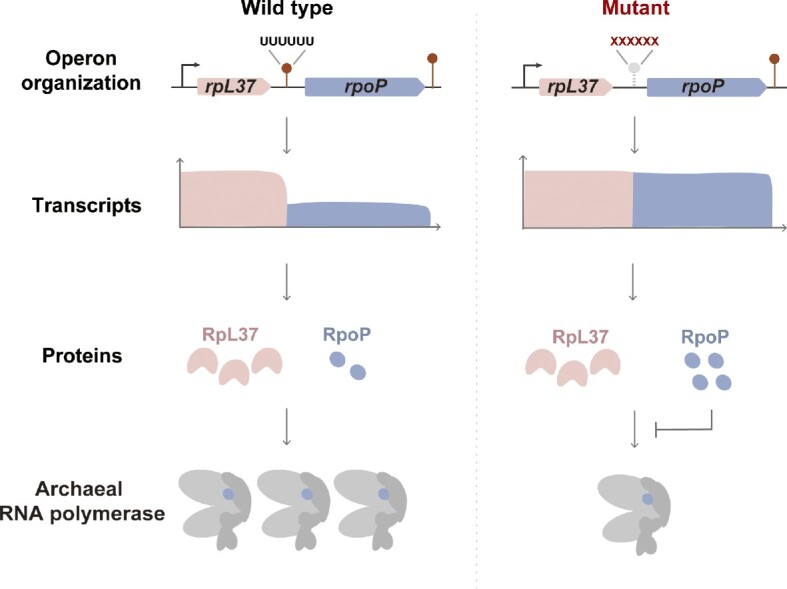

## INTRODUCTION

The pioneering study of Jacob and Monod in 1961 generated the operon hypothesis (1–3), which states that genes involved in related functions are generally organized within operons and co-transcribed, providing an efficient means to synchronously coregulate transcription of multiple genes and produce equal amounts of subunits for assembling complexes (2,4–7). The presence of operons also distinguishes the genomic organization of prokaryotes from that of eukaryotes. While differential protein production is commonly found for genes within operons encoding multi-subunit complexes, such as the ribosome, secretion machinery, ATP synthase, and antiviral defense systems (8–11), the mechanisms involved are not well documented. Selective mRNA processing was found to regulate the differential production of the cellulase subunits in the *Clostridium cellulolyticum* operon (12) and ribosome subunits in an archaeal operon (13). Based on informatic analyses of bacterial genomes, transcriptomes and ribosomal profiling data, differential translation efficacy depending mainly on mRNA secondary structure and codon usage was presumed to be a key determinant in differential protein production from genes within bacterial operons to maintain appropriate stoichiometries of proteins required for formation of complexes (14–18).

Using SMRT-Cappable-seq, the full-length transcriptomic map of *Escherichia coli* was recently obtained. It not only accurately defined genome-wide transcription units (TUs) and operons, but also revealed extensive transcription read-through at over 40% of the transcription termination sites (TTSs), which especially complicated the operon transcription profiles (19). Extensive transcription read-through was also more recently reported in several other bacteria (16,20), indicating the complexity of the transcriptional landscape in bacteria. In previous study, using Term-seq to sequence the genome-wide TTSs, we found that the majority of operons in the methanogenic archaeon *Methanococous maripaludis*, one representative of the third life form Archaea, exhibited incomplete termination at the TTSs. Depending on the terminator strength, only 30–80% terminations were observed for the majority of TTSs. Most terminations depended on the terminator strength (21) and also a newly discovered transcription termination factor aCPSF1. As this protein was essential, depletion of the cellular levels of aCPSF1 caused a genome-wide reduction in transcription termination, severe growth inhibition, and generation of a chaotic transcriptome (21,22). TTSs were also observed in the intergenic regions (IGRs) within some putative operons in the Term-seq map, and Illumina-seq data detected different transcription levels of the genes within operons. This suggested that transcription termination might occur within many operons and could exert a regulatory role in fine-tuning expressions of the operon genes.

This study extends the above observations. The combination of PacBio-seq, dRNA-seq, Term-seq and Illumina-seq of the *M. maripaludis* transcriptome thoroughly deciphered the operon organization and surprisingly found that the internal operon intergenic terminators, here named as ioTTS terminators, are widely distributed. The ioTTS terminations were found to be functional, causing reduced transcription of down-stream genes and reduced production of the encoded proteins. Mutation of representative ioTTS terminators increased levels of the transcripts and encoded proteins of the downstream genes. Furthermore, these mutations often severely inhibited growth, demonstrating the importance of ioTTS terminations in overall cellular fitness. Of special interest, mutation of the ioTTS terminators in ribosomal protein-RNA polymerase (RP-RNAP) operons also decreased the cellular RNAP complex abundances and the transcription and translation velocities, demonstrating a key role in controlling production of the RNAP complex. Given that similar RP-RNAP operons are ubiquitous in archaea, this work suggests that ioTTS termination could be a novel and potentially ancient mechanism for tuning differential expression of the operon genes in archaea.

## MATERIALS AND METHODS

### Strains, plasmids and culture conditions

Strains and plasmids used in this study are listed in Supplementary Table S1. *M. maripaludis* and its derivatives were routinely cultured in anaerobic McF medium under a gas phase of N_2_/CO_2_ (80:20) as previously described (23). As aCPSF1 was depleted to a low abundance (20% compared to the wild type) at 22°C and much lower than the abundance at 37°C (∼50%), the transcriptomic analysis and the experiments with the aCPSF1 depletion strain were all performed at 22°C unless specially indicated otherwise. These were the same conditions used for the previously collected Term-seq, differential RNA-seq (dRNA-seq) and Illumina-seq data (21,22). For solid medium, 1.5% agar was added. For Fe-limited growth, the concentrations of Fe (II) were adjusted by varying the level of ammonium iron(II)sulfate in McF medium. Growth was monitored by determining the optical density at 600 nm (OD_600_). Unless indicated otherwise, 2.5 μg ml^−1^ puromycin was added for genetic selections and to maintain recombinant plasmids in *M. maripaludis*. *E. coli* strains were routinely grown in LB Miller broth at 37°C with shaking at 200 rpm. When needed, final concentrations of antibiotics and supplements used were ampicillin at 100 μg ml^−1^, kanamycin at 50 μg ml^−1^, isopropylthiogalactoside (IPTG) at 0.1 mM.

### PacBio sequencing of the transcriptome

Long reads of the reverse transcribed *M*. *maripaludis* mRNAs were sequenced on the PacBio platform as described previously (19). Briefly, total RNA was extracted from mid-exponential *M. maripaludis* cultures using TRIzol^TM^ reagent (Invitrogen) as described previously (13,22). The extracted RNA had an RNA integrity number (RIN) determined by Bioanalyzer (Agilent) ≥9.0. Then 5 μg of the extracted RNA was capped through a capping reaction, and then a poly-A tail was added using *E. coli* Poly(A) Polymerase (New England Biolabs). rRNA was depleted using Ribo-Zero™ rRNA Removal Kit (Epicentre). The capped and tailed RNA was purified using AMPure beads and eluted in TE buffer, and the full-length primary transcripts were further enriched using streptavidin magnetic beads (New England Biolabs). Polyadenylation (A-tailing) ensured priming by the poly dT primer at the 3′ end of transcript and reverse transcription of the first cDNA strand. PolyG was then added to the cDNA 3′end for second-strand synthesis using Terminal Transferase (TdT, New England Biolabs). Unenriched full-length transcripts prepared without the capping reaction and streptavidin enrichment were used to construct a control library for verifying the positions and abundances of transcription start sites (TSSs) and transcription termination sites (TTSs) of operons. Library construction and PacBio-sequencing were performed at Frasergen Bioinformatics Technology Co., Ltd (Wuhan, China). As described previously (19), the PacBio-sequencing reads were filtered to remove chimeras and trimmed for 3′end polyA and 5′end polyC prior to mapping on the *M. maripaludis* genome.

### Operon identification by bioinformatic analysis of multiple transcriptomic data

Operons were identified based on the mapping of the PacBio reads in combination with data of the TSSs and TTSs that were previous identified at single-base resolution via dRNA-seq and Term-seq (22), respectively. Transcription units (TU) containing at least a TSS at the 5′ end, a TTS at the 3′ end, and a continuous coverage by a PacBio read for the full length of the RNA was defined as an operon. In addition, a downstream gene encoded on the same strand must possess its own TSS. The transcript boundaries and abundances were then verified by data from the previous Illumina-sequenced transcriptome (22). TSSs and TTSs identified in the intergenic regions within an operon were named the internal operon TSSs (ioTSSs) and internal operon TTSs (ioTTSs), respectively. Using WebLogo v2.8.2, the terminator motifs preceding the ioTTSs were analyzed from 36 nt upstream to 1 nt downstream (24) (Dataset S1). The ioTTS-based differential expression ratio (TDER) of operon genes was calculated from the ratio of the transcript abundances of the ioTTS-upstream genes divided by the abundances of the downstream genes. TDERs of >2, <2 & >1.5 and <1.5 were defined as having high-, mid-, and low significance, respectively. Transcription termination efficacy (TTE) of an ioTTS terminator was defined as 1-TDER^−1^ in the *M. maripaludis* wild-type transcriptome. aCPSF1 dependency of TDER is (TDER of the aCPSF1 depletion strain ▽*aCPSF1*)/(TDER of the wild-type strain) (Dataset S2).

### Northern blot assay

Northern blot assays were performed as previously described (22). Briefly, total RNA was extracted from the mid-exponential cells using TRIzol™ reagent (Invitrogen). After quantification using a Nano photometer spectrophotometer (Implen), RNA was denatured at 65°C for 10 min in loading buffer containing 95% (v/v) formamide, and 5–10 μg was loaded in each lane of a 6% polyacrylamide gel with 7.6 M urea. Electrophoresis was performed in 1× TBE buffer. A single-stranded RNA (ssRNA) ladder (New England Biolabs) served as a size marker. After separation, RNAs were transferred onto Hybond-N^+^ membranes (GE Healthcare) by electroblotting and crosslinked to the membrane using UV. Next, membranes were prehybridized at 42°C in prehybridized buffer (5 × SSC, 5 × Denhardt′s, 50% v/v formamide deionized, 0.5% w/v SDS, 200 μg ml^−1^ pre-denatured Salmon sperm DNA)for 4 h, followed by hybridization for 12 h with 2–10 pmol of biotin-labeled DNA probes listed in Supplementary Table S4. After three rounds of washing for 10 min in 1×, 0.2× and 0.1× SSC–0.1% SDS solutions, band signals were visualized using a chemiluminescent nucleic acid detection module (Thermo Scientific) according to the manufacturer's protocol.

### Quantification of transcript abundances by quantitative RT-PCR (qRT-PCR)

Total RNA was extracted from the mid-exponential cells as described above, and 500 ng of RNA was used to generate complementary DNAs using ReverTra Ace® qPCR RT Master Mix with gDNA Remover (Toyobo). Quantitative PCR amplifications were performed with Mastercycler eprealplex2 (Eppendorf AG, Hamburg, Germany) and a parallel amplification without RT reaction was used as a control to assess the absence of DNA in the RNA samples for each gene (Supplementary Figure S1). The standard curve for each gene was generated using 10-fold serially diluted PCR products as template. The primers used are listed in Supplementary Table S3. Transcript abundances were then normalized by comparison to the abundance of the 16S rRNA. All assays were performed on triplicate samples and repeated at least three times.

### Recombinant protein purification

To obtain the recombinant *M. maripaludis* proteins in *E. coli*, the respective open reading frames (ORFs) for the His-tagged RpoP, Rpl37Ae, Rpl21e and AfuA and GST-tagged RpoF and AfuC proteins were cloned into the expression vector pET28a or pGEX 4T-1 via stepwise Gibson assembly using ClonExpress MultiS One Step Cloning Kit (Vazyme). The expression plasmids were then transformed into *E. coli* BL21(DE3)pLysS, which was cultured at 37°C in LB broth containing 50 μg ml^−1^ kanamycin or 100 μg ml^−1^ ampicilin. When the OD_600_ reached 0.6–0.8, 0.1 mM isopropyl-b-d-thiogalactoside (IPTG) was added. After 16 h at 22°C, cells were harvested by centrifugation and stored at -80°C. The harvested cells were resuspended in binding buffer (for his-tagged proteins: 0.5 M NaCl, 20 mM imidazole, 5% v/v glycerol, 20 mM HEPES, pH 7.5; for GST-tagged proteins: 0.5 M NaCl, 26 mM Tris-base, 0.97 mM DTT, 5% v/v glycerol, pH 7.5) and lysed by sonication, and the supernatant was collected by centrifugation. The his-tagged or GST-tagged recombinant proteins were purified from the supernatant by passage through a His-Trap HP column or a GSTrap HP column (GE Healthcare) according to the manufacturer's protocol (25). Purified proteins were analyzed by SDS-PAGE, and the protein concentration was determined using a BCA protein assay kit (Thermo Scientific).

### Western blot assays

Western blots were performed to determine the cellular protein abundances in the wild-type and genetically modified strains of *M. maripaludis*. The polyclonal rabbit antisera against the purified proteins were generated by MBL International Corporation. Mid-exponential cells of *M. maripaludis* were harvested and resuspended in Lysis Buffer [50 mM Tris–HCl (pH 7.5), 150 mM NaCl, 10 (w/v) glycerol, 0.05% (v/v) NP-40 detergent] and lysed by sonication. The cell lysate was centrifuged at 14,000 g for 15 min at 4°C, and proteins in the supernatant were separated on 15% SDS-PAGE and transferred to a nitrocellulose membrane. The antisera to the proteins were diluted 1:5000. A horseradish peroxidase (HRP)-linked secondary conjugate at a 1:5000 dilution was used for the immunoreaction. Immune-active bands were visualized by an Amersham ECL Prime western blot detection reagent (GE Healthcare). For quantifying the cellular content of a protein, cell-free extract of *M. maripaludis* at indicated amounts was electrophoresed on SDS-PAGE synchronously with loading the corresponding purified recombinant proteins from *E. coli* as references. Density of each protein band was quantified from photographs using ImageJ software, and the cellular abundance of a protein was calculated by comparison to the respective recombinant protein.

### Construction of strains with mutations in the ioTTS terminators

The *M. maripaludis* strains with ioTTS terminator mutations were constructed using the recently developed CRISPR-Cas9 genome-editing toolkit (26). Based on pMEV4-Cas9 that carries the *Streptococcus pyogenes* Cas9 and a small guide RNA (sgRNA) sequence under control of constitutive *M. maripaludis* promoters, the plasmids for ioTTS terminator mutations (Supplementary Table S1) were constructed in three steps. First, a sgRNA sequence of 20-bp targeting the terminator region was designed and inserted into the sgRNA expression region of pMEV4-Cas9. Second, a donor DNA carrying the upstream and downstream homologous arms flanking the sgRNA targeting region was inserted into the donor region of pMEV4-Cas9. Third, the sequence for the mutated ioTTS terminator was inserted into the donor sequence by stepwise Gibson assembly using the primers listed in Supplementary Table S2. PCR amplification was performed using the high-fidelity KOD-plus DNA polymerase (TOYOBO, Japan). DNA fragments and the amplified plasmid backbone were ligated by stepwise Gibson assembly using ClonExpress MultiS One Step Cloning Kit (Vazyme). The sequences of the constructed plasmids were confirmed by DNA sequencing prior to PEG-mediated transformation into *M. maripaludis*. Positive mutants were selected first by puromycin resistance to obtain the transformants and then 8-azahypoxanthine resistance to remove pMEV4-Cas9 plasmid as described previously (27).

### Construction of RpoP and RpoF overexpression strains

The RpoP and RpoF overexpression strains in *M. maripaludis* were constructed using the expression plasmid pMEV4 that carries the puromycin resistance gene *pac* and the terminator T_MMP1559_. DNA fragments containing the ORF regions of *M. maripaludis rpoP* or *rpoF* were PCR amplified using primers listed in Supplementary Table S2 and then inserted into pMEV4 under control of the constitutive promoter of *MMP0386* through Gibson assembly to obtain plasmids P*_MMP0386_*-*rpoP*-*pac*-T_MMP1559_ and P*_MMP0386_*-*rpoF*-*pac*-T_MMP1559_ (Supplementary Table S2). Similarly, a pMEV4 plasmid carrying the mCherry gene, P*_MMP0386_*-Mcherry-*pac*-T_MMP1559,_ was constructed as a control. The plasmids were each transformed into *M. maripaludis* via the PEG-mediated method to produce the overexpression strains pMEV4-P_0386_-*rpoP*, pMEV4-P_0386_-*rpoF* and pMEV4-P_0386_-*mcherry* (Supplementary Table S1) through puromycin selection.

### Overall transcription rate estimation by ^3^H-uridine labeling

The relative overall transcription rate was compared by determining the rate of ^3^H-uridine incorporation into the exponential cultures of *M. maripaludis* grown at 22°C. Portions of exponentially growing cultures at OD_600_ of ∼0.45, 600 μl, were collected at 0, 0.2-, 0.5-, 1-, 1.5- and 2-min post-addition of [5,6–^3^H]-Uridine (PerkinElmer) at a final concentration of 105 μCi/ml. Total RNA was extracted using TRIzol^TM^ reagent (Invitrogen) and dissolved in 700 μl Ecoscint A (National Diagnostics). ^3^H isotope incorporation was determined by liquid scintillation counting (Perkin Elmer). Relative overall transcription rate was calculated from the linear increase in cpm incorporation with time.

### Nascent protein synthesis determined by puromycin incorporation

Puromycin incorporation was employed to determine the abundance of nascent proteins (28). Puromycin (catalog no. P8833; Sigma) was added to a final concentration of 1% (w/v) to exponentially growing cultures at OD_600_ = 0.5. After 10 min at 37°C, cells were harvested, resuspended in Lysis Buffer, and lysed by sonication. Total protein in cell-free extracts (CFE) was quantified using a BCA protein assay kit (Thermo Scientific). Five microgram of CFE protein was western-blotted to quantify the puromycin incorporated using the monoclonal anti-puromycin antibody at a 1:10 000 dilution (Millipore Company, Darmstadt, Germany) and HRP-conjugated, anti-mice secondary antibody (Abmart) at a 1:2000 dilution.

### Size exclusion chromatography

Cells of *M. maripaludis* in the exponential growth phase at OD_600_ of ∼0.45 were harvested and resuspended in Lysis Buffer, lysed by sonication, and centrifuged at 12,000 g for 30 min at 4°C. The supernatant was incubated at 37°C for 30 min with and without treatment of DNase I (100 U) and RNase A (100 U). A total of 500 μg of the pretreated supernatant was fractionated through the size exclusion chromatography on a Superdex 200 10/300 GL column (GE Healthcare), and the Mr of each fraction was estimated by gel filtration molecular weight markers (Kit No.: MWGF1000 of Sigma-Aldrich). Finally, each fraction was collected for protein identification by western blotting.

## RESULTS

### Internal-operon termination sites (ioTTSs) and internal-operon start sites (ioTSSs) are common in *M. maripaludis*

**Figure 1.**
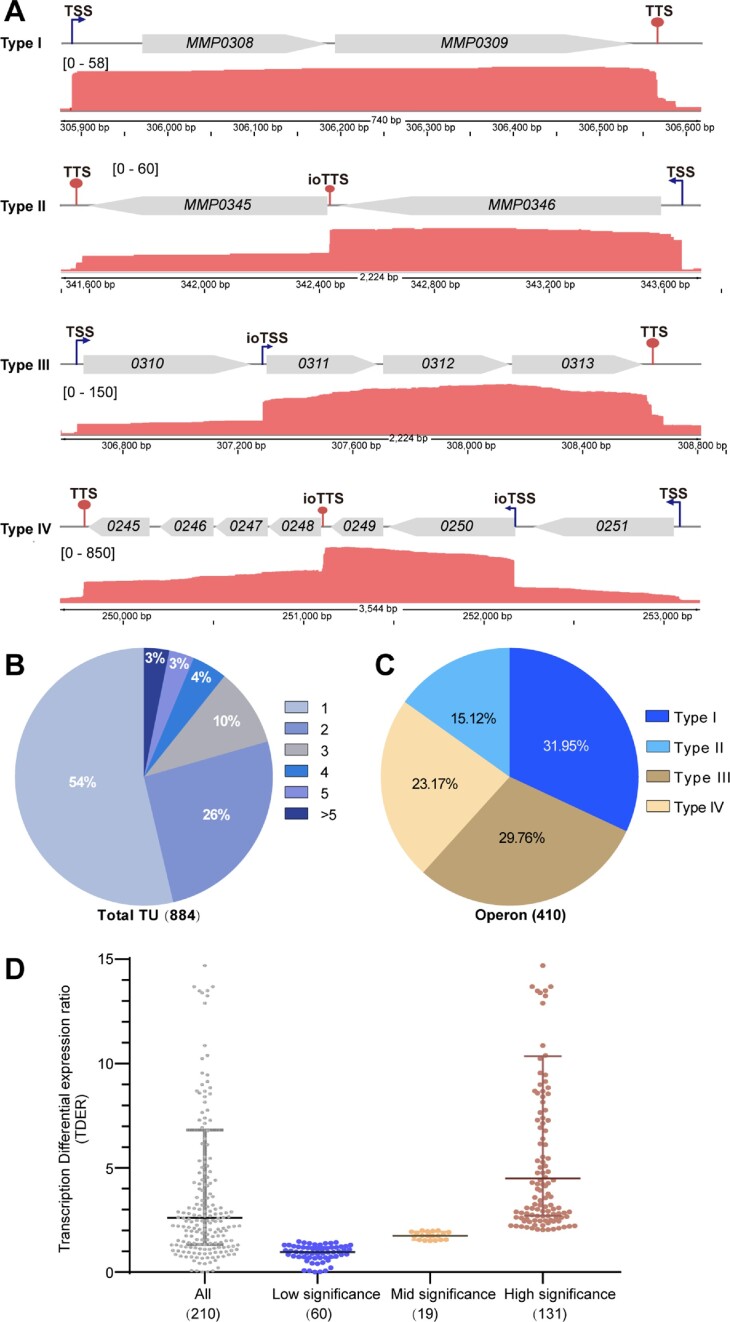
Overview of the *M. maripaludis* operons determined via a combination of PacBio-based full-length transcript sequencing with dRNA-seq, Term-seq and Illumina-seq. (**A**) Representative PacBio-seq maps of the four operon types in *M. maripaludis*. TSS and TTS indicate transcription start and termination site of an operon, respectively; ioTSS and ioTTS indicate the internal operon TSS and TTS between intergenic genes, respectively. Operon organizations are shown at the top, numbers in brackets show the scale for the number of PacBio-seq reads, and numbers at the bottom indicate the regions of the genome map. (**B**) Number of genes in TUs. (**C**) Percentages of each type in the 410 operons. (**D**) The ioTTS-based differential expression ratios (TDER) in the 210 ioTTS-containing operons. Ratios <1.5, >1.5 & <2 and >2 are considered possessing low-, mid- and high-biological significance, respectively. *T*-test statistical analyses showing the *P* values for each ioTTS TDER are listed in Dataset S2.

PacBio-seq enabled mapping the full length of transcripts in *M. maripaludis*. When integrated with previously published dRNA-seq data of transcription start sites (TSSs) (29), Term-seq data of transcription termination sites (TTSs) (21,22), and Illumina-seq data of transcript abundances (22), the operon map of *M. maripaludis* was characterized at single-base resolution (Figure 1A). In total, 884 transcriptional units (TUs) were identified among the total of 1722 coding genes, in which half of the TUs (410/884) were operons, ie. comprising more than one gene (Figure 1B and C). The operon length was positively correlated with the number of genes, and the median length was 1794 nt or close to the median length of operons composed of two genes (Supplementary Figure S2A). Remarkably, internal operon TSSs (ioTSSs) and TTSs (ioTTSs) were found in the intergenic regions (IGRs) within 53% and 38% operons, respectively. Four operon types were identified (Figure 1A and C). Type I did not contain either ioTSSs or ioTTSs. Type II contained at least one ioTTS. Type III contained at least one ioTSS. Type IV contained both ioTTS and ioTSS (Dataset S1). The median lengths of type II and III operons were also longer than that of type I operons (Supplementary Figure S2B), and the longer operons possessed more ioTTSs or ioTSSs (Supplementary Figure S2C and D).

### ioTTSs correlated with differential expression of upstream and downstream genes

ioTTSs were found in 38% (157/410) of operons. The ioTTSs were characterized by their transcription differential expression ratios (TDERs) of the flanking genes or the transcript abundance of the upstream gene divided by that of the downstream gene. TDERs >1.5 were considered biologically significant, and TDERs >2 were considered as highly significant biologically (see below). For the operons containing ioTTSs, 72% (150/210) of the TDERs possessed mid- or high-significance (Figure 1D). Thus, most of the ioTTSs were functional, suggesting that they are the cause for the uneven transcription of the flanking genes within the operons.

In *M. maripaludis*, transcription termination depends upon the general transcription termination factor aCPSF1 and occurs at a *cis-*element with a polyuridine (polyU)motif (21,22). To determine if the mechanism of ioTTS termination resembles to that at the end of TUs, the role of aCPSF1 and terminator motif were examined. The TDERs for many of the ioTTSs were much lower in the mutant ▽*aCPSF1*, which possessed only low levels of aCPSF1 (20% of wild type) by growing at 22°C but not 37°C (Supplementary Figure S3A). Similarly, terminators with a higher termination efficiency had a higher dependency on aCPSF1 than the weak terminators (Supplementary Figure S3C). These observations were consistent with an important role for aCPSF1 at ioTTSs. In addition, the motifs preceding the ioTTSs with biologically significant TDERs featured pronounced polyU-tracts at the termination sites (Supplementary Figure S3B). In summary, termination at ioTTSs also depended on the presence of both polyU tracts and the transcription termination factor aCPSF1, similar to the requirement for a trans-acting factor and *cis-*element for archaeal transcription termination at the end of TUs (21).

**Figure 2.**
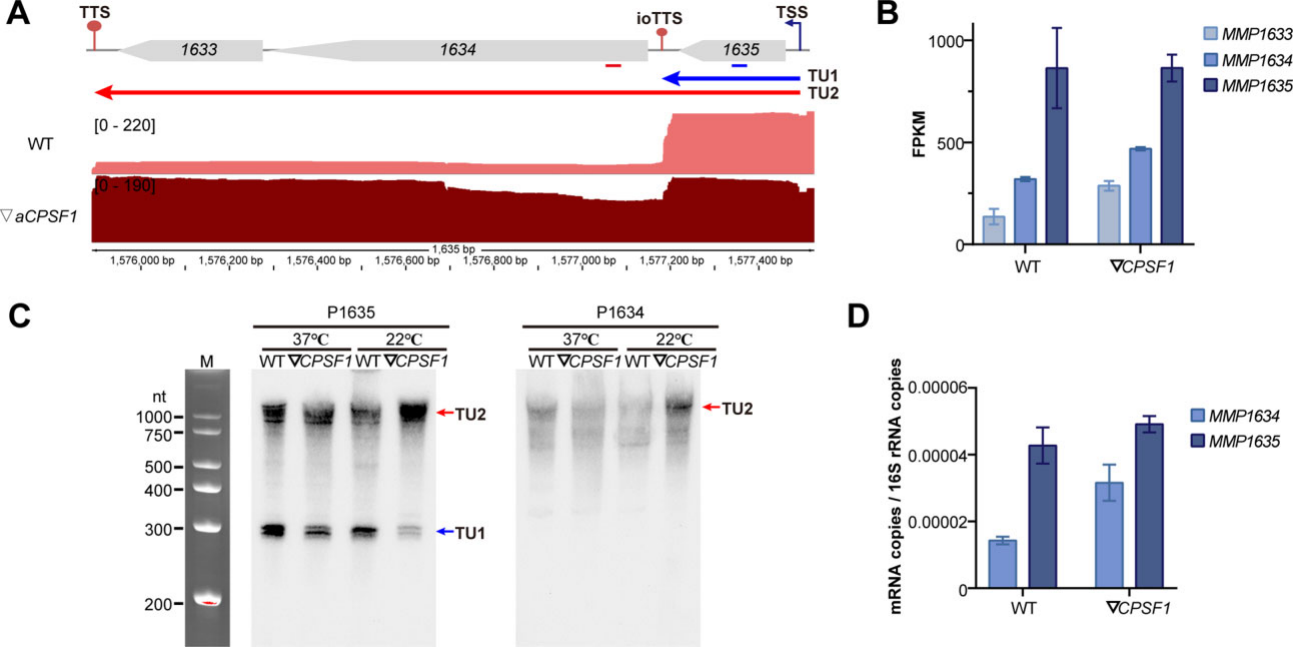
Experimental verification of transcription termination at the ioTTS within the *MMP1635-1633* operon in *M. maripaludis*. (**A**) PacBio-seq schematic displayed transcript reads of *MMP1635* and *MMP1634-1633* in the wild type (WT) and aCPSF1-depletion mutant (▽*aCPSF1*). Predicted transcription units (TUs) are shown as horizontal arrows. Operon organized genes are shown as bullets, and TSS, TTS and ioTTS are indicated. Numbers in brackets show the scale for the number of PacBio-seq reads, and numbers at the bottom indicate the regions of the genome map. (**B**) A bar diagram shows the abundances for the Illumina-sequenced transcripts of *MMP1635, MMP1634* and *MMP1633* in the wild type and the ▽*aCPSF1* mutant. Error bars indicate one standard deviation of the averages. (**C**) Northern blot of the transcripts of the *MMP1635-1633* operon in the wild type and ▽*aCPSF1* strains cultured at 37°C and 22°C. Probes targeting *MMP1635* and *MMP1634* hybridized to the transcripts indicated by blue and red lines in (A). Transcription termination defects were observed more severely at 22°C as expression of aCPSF1 was depleted to ∼20% of wildtype 22°C compared to ∼50% of wildtype at 37°C (22). A ssRNA marker is shown at the left. (**D**) Transcript abundances of *MMP1635* and *MMP1634* assayed by qRT-PCR. The primers used are listed in Supplementary Table S3. The results are averages and standard deviations of triplicate cultures. Unless indicated otherwise, all cultures were grown at 22°C.

To confirm these general properties of ioTTSs, three operons each consisting of ≥ 2 genes and one ioTTS were selected. PacBio-seq identified one ioTTS in the trigenic operon *MMP1635-1633* that encodes a thioredoxin/glutaredoxin homolog, DsrE-like protein, and a conserved hypothetical protein. A sharp decrease in transcription of *MMP1634-1633* immediately downstream of the ioTTS was observed, and this decrease was greatly alleviated in the ▽*aCPSF1* strain (Figure 2A). Similar results were found by Illumina-seq. *MMP1634* had a 2.7-fold and 1.8-fold lower transcription abundance than *MMP1635* in the wildtype and ▽*aCPSF1* strains, respectively (Figure 2B). Northern blotting confirmed these results. A probe targeting *MMP1635* detected both the expected three-cistron transcript > 1000 nt in length and the monocistronic transcript of *MMP1635* in the wildtype strain. In contrast, depletion of *aCPSF1* resulted in an increase of the three-cistron transcript and diminution of the monocistronic transcript (Figure 2C). Similarly, the *MMP1634* probe only detected the three-cistron transcript, whose abundance increased following growth of the mutant at 22°C, a condition where the levels of aCPSF1 were reduced to 20% of the wildtype (22). qRT-PCR also detected a 3-fold and 1.6-fold lower abundance of the transcripts downstream of the ioTTS in the wildtype and ▽*aCPSF1* strains, respectively (Figure 2D). Differential transcription of the ioTTS flanking genes was also displayed for other operons (Supplementary Figure S4) and similarly verified in another two operons, *MMP0290-0291* (Supplementary Figure S5) and *MMP1190-1189* (Supplementary Figure S6). Collectively, these experiments verified the partial termination at ioTTSs by multiple experimental methods at multiple representative operons.

### Role of ioTTS termination in differential expression of genes within operons encoding protein complexes

**Figure 3.**
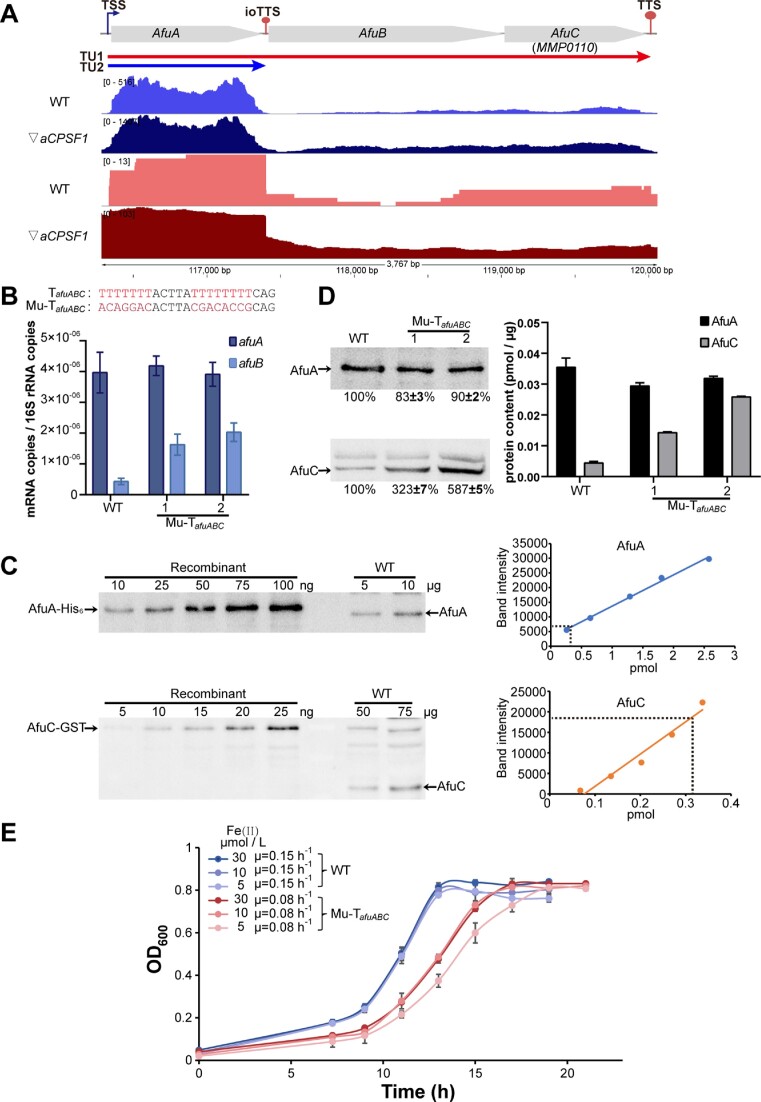
Importance of ioTTS termination in the expression of the AfuABC transporter subunits. (**A**) Illumina-seq (blue, dark blue) and PacBio-seq (red, dark red) maps of the transcription profiles of the *afuABC* operon in the wild type (WT) and ▽*aCPSF1* mutant. Unless indicated otherwise, all cultures were grown at 22C. The transcriptional units (TUs) are indicated by the colored arrows at the top. TSS, ioTTS, TTS, TU, and numbers in bracket and at bottom are the same as in Figure 1A. (**B**) qRT-PCR to quantify the *afuABC* transcription in the wild type and the Mu-T*_afuAB_* mutant. In this mutant, the ioTTS*_afuAB_* between *afuA* and *afuB* was mutated by replacement of the T-rich tracts as shown at top of panel B. Transcript abundances were measured in the wild type and two replicate colonies of the ioTTS terminator mutant. (**C**) Western blot assay of AfuA and AfuC protein contents in wild type. The anti-AfuA and anti-AfuC polyclonal antibodies were used to detect the AfuA and AfuC proteins in the wild type. The standard curve of recombinant protein (right) was calculated from the band intensities (left). (**D**) Western blot assay of AfuA and AfuC proteins in the wild type and two replicate colonies of the Mu-ioTTS*_afuAB_* mutant (left), and the calculated protein contents (right). (**E**) Growth of the wild type (WT) and a representative Mu-T*_afuABC_* mutant at different Fe(II) concentrations. Triplicate experiments were performed, and averages and standard deviations of the OD_600_ are shown.

In *M. maripaludis*, a number of operons encode multisubunit enzymes and other protein complexes. In some cases, the stoichiometries of the subunits differ, and ioTTS termination could play a role in the differential expression of their genes. To explore the significance of ioTTS termination in expression of operons encoding protein complexes, the operon *afuABC* encoding the Afu ABC transporter for Fe(II) uptake was selected. An ioTTS, assigned as ioTTS*_afuAB_*, was found in the IGR between *afuA* and *afuB*. While, no evidence for ioTSS or antisense RNA was detected in this operon. Illumina-seq and PacBio-seq detected a higher abundance of transcripts for *afuA* than *afuBC* (Figure 3A), and qRT-PCR assayed >9-fold higher levels of *afuA* transcripts than those of *afuB* in the wild type (Figure 3B). Quantitative western-blotting also found 8-fold higher levels of the proteins AfuA than AfuC (0.0354 pmol/μg vs. 0.0044 pmol/μg total cell proteins) (Figure 3C and D). In contrast, mutants where the ioTTS*_afuAB_* terminator polyU-tract was disrupted using the CRISPR-Cas9 toolkit developed for *M. maripaludis* (26) possessed significantly elevated levels of *afuB* transcripts (Figure 3B) and a 3−5-fold increase in the AfuC but not AfuA protein content (Figure 3C). In addition, the mutant grew more poorly than the wild type, especially at low concentrations of Fe (II), suggesting that the iron transporter in the mutant was functionally defective (Figure 3E). Thus, the ioTTS was necessary for the correct expression of the genes in the *afuABC* operon, the resulting differential abundances of the encoded subunits, and presumably the correct or optimal assembly of the Afu ABC transporter, as reflected in the poor growth of the mutant.

To further evaluate the physiological role of ioTTS terminators, ioTTSs were inserted into an operon that did not have one. The 10-gene operon encoding the ATP synthase complex was identified by both Illumina and PacBio transcriptome sequencing as lacking an ioTTS (Supplementary Figure S7A). Furthermore, transcript abundance was the same in the wild type and ▽*aCPSF1* mutant, confirming that no internal transcription termination occurred within the operon. Insertion of a strong terminator from *MMP0204* (T1) or a medium-strength terminator from *MMP0229* (T2) into the IGRs of *atpK*-*atpE* and *atpF*-*atpA* reduced transcription of the ioTTS downstream genes and also disrupted the coordinated transcription of the ten genes in the operon (Supplementary Figure S7). Growth of the *M. maripaludis* mutants was also reduced, compared from 0.15 h^−1^ of the wild type to 0.07 h^−1^ and 0.08 h^−1^ of the T1 and T2 terminator mutants, respectively (Supplementary Figure S7D). In conclusion, ioTTSs are not only necessary for the proper expression of the genes in some operons, but also they are detrimental when positioned incorrectly in the intergenic regions of other operons.

### ioTTS termination controls expression of the ioTTS downstream RNAP subunits in RP-RNAP operons

**Figure 4.**
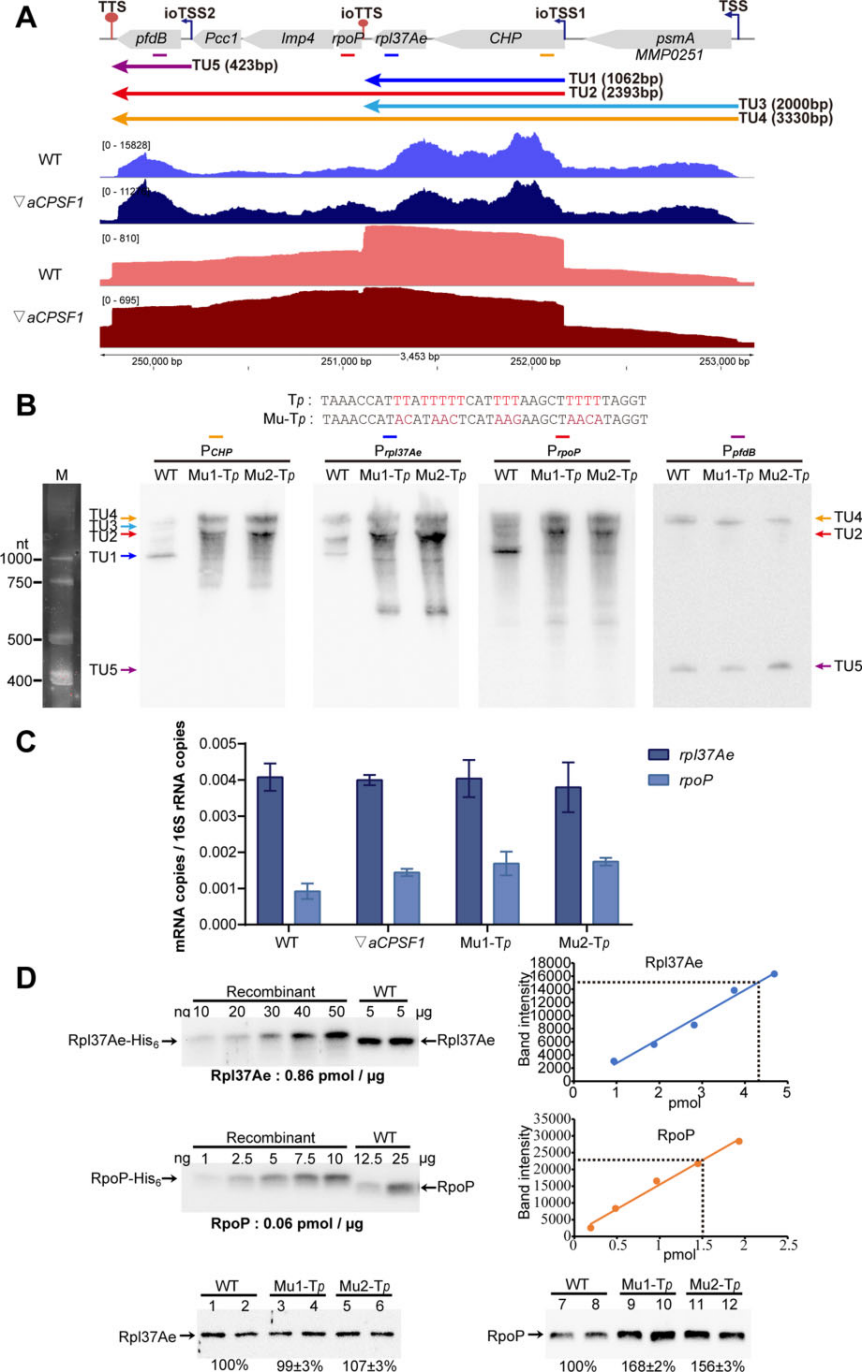
Role of ioTTS termination in controlling expression in the *rpl37Ae-rpoP* operon. (**A**) Illumina-seq (blue, dark blue) and PacBio-seq (red, dark red) maps of the transcription profiles of the *rpl37Ae-rpoP* operon in the wild type (WT) and ▽*aCPSF1* mutant. Colored arrows represent transcription units (TUs) predicted from transcriptomic sequencing data. TSS, ioTTS, TTS, ioTSS, TU and numbers in bracket and at bottom are the same as indicated in Figure 1A. (**B**) Northern blots assays of *rpl37Ae-rpoP* transcription in the wild type and the Mu-T*_p_* mutant. The ioTTS*_rpl37Ae/rpoP_* terminator (T*_P_*) between *rpl37Ae* and *rpoP* was mutated by replacing the T-rich tracts as shown at the top of (A) to obtain the terminator mutant Mu-T*_p_* Using four DNA probes whose locations are indicated by the colored bars in (A) and whose sequences are in Supplementary Table S4, the transcription profiles of the *rpl37Ae-rpoP* operon in the wild type and two colonies of Mu-T*_p_* mutant were assayed by northern blotting. TUs were assigned by reference to the ssRNA molecular weight markers (M) at left. (**C**) Quantification of *rpl37Ae* and *rpoP* transcript abundance in the wild type, the ▽*aCPSF1* mutant, and two replicate colonies of mutant Mu-T*_p_* Primers targeting *rpl37Ae* and *rpoP* are listed in Supplementary Table S3. Triplicate cultures were assayed, and averages and standard deviations are shown. (**D**) Western blot assays of the cellular Rpl37Ae and RpoP contents in wild type using anti-Rpl37Ae and anti-RpoP polyclonal antibodies. Left-hand panels are the gels used to generate the standard curves with recombinant proteins and wild-type extract. The calculated protein concentrations in extract are the right-hand panels. Bottom panels are the relative concentrations of Rpl37Ae and RpoP proteins in the wild type and two colonies of Mu-T*_p_* mutant. Three cultures of each of the two colonies were assayed, and one representative result is shown here. All cultures were grown at 22C.

*M. maripaludis* possesses an RNA polymerase (RNAP) homologous to the eukaryotic RNAP II and composed of 12 subunits and encoded in five operons (Supplementary Figure S8). One operon, *rpoHB2B1A1A2*, encodes the four catalytic subunits and an auxiliary subunit. The genes *rpoDLNP*, encoding four assembly subunits, and *rpoEFK*, encoding three auxiliary subunits are located in operons mostly comprising ribosomal protein (RP) genes, forming RP-RNAP operons. Because the levels of the RPs and RNAP proteins are different, differential transcription of the genes might be necessary for correct protein production. Interestingly, in these operons the RNAP genes are mostly located downstream of a RP gene, and an ioTTS separates them (Supplementary Figure S8). Accordingly, higher transcript abundances were found for the upstream RP than the downstream RNAP genes (Figure 4A, Supplementary Figures S9A and D, and S10A, and Dataset S1). Northern blotting and qRT-PCR verified the differential transcriptions of the downstream and upstream genes, and this difference was reduced in the ▽*aCPSF1* mutant (Figure 4 and Supplementary Figure S9). Thus, termination at the ioTTS appeared to be at least partly responsible for the correct expression of the genes in the RP-RNAP operons.

To examine ioTTS termination further, a mutation was generated in the ioTTS*_rpl37/rpoP_* of the operon containing *rpl37Ae* and *rpoP* (Figure 4B). The ioTTS*_rpl37/rpoP_* terminator polyU-tract was replaced with non-U sequence to generate the mutant Mu-T*_p_* (Figure 4B). Northern blotting with four probes targeting the genes downstream of the two ioTSSs and flanking ioTTS*_rpl37/rpoP_* detected five TUs of different lengths in wild type and the Mu-T*_p_* mutant, with a two-cistron transcript TU1 including *rpl37Ae* and a six-cistron transcript TU2 including *rpl37* and *rpoP* being most abundant. In the Mu-T*_p_* mutant, the abundance of TU1 was markedly reduced while that of TU2 was elevated. In addition, the 7-cistron TU4, which comprises all the genes in this operon, was also detected in the mutant (Figure 4B). Similarly, qRT-PCR detected a 4.5-fold higher level of transcripts of *rpl37Ae* than *rpoP* in the wild type, but only 2-fold and 2.7-fold higher in the Mu-T*_p_* and ▽*aCPSF1* mutants, respectively (Figure 4C). Likewise, western blotting detected 14.3-fold higher levels of Rpl37Ae (0.86 pmol/μg) than RpoP (0.06 pmol/μg) in the wild-type strain. However, 1.6-fold higher levels of RpoP were detected in the Mu-T*_p_* mutant than in wild type, which consequently reduced the differential ratio of Rpl37Ae versus RpoP from 14.3-fold to 8.9-fold (0.88 pmol/μg vs. 0.097 pmol/μg). Therefore, these results confirmed the role of the ioTTS in reducing both transcription and protein production of downstream genes within the RP-RNAP operon (Figure 4D).

Involvement of ioTTS termination in expression of RP and RNAP genes was also investigated in a tri-cistron operon containing *rpl21e-rpoF*. This operon contains ioTTS*_rpl21/rpoF_* in the *rpl21e-rpoF* IGR and two ioTSSs upstream of *rpl21e* (Supplementary Figure S10A). Using probes targeting each gene, northern blotting detected five TUs, with the 3-cistronic TU1, TU4 containing *rpl21e-rpoF*, and TU5 containing only *rpl21e* being abundant in wild type (Supplementary Figure S10B). Following mutation of the ioTTS*_rpl21/rpoF_*, abundances of the TU2 and TU4 containing *rpl21e-rpoF* were elevated while the abundances of TU5 and TU3, which contained only *rpl21e*, were reduced. Thus, there appeared to more read-through of the ioTTS in the mutant. Similarly, qRT-PCR found 2.5-fold more transcripts of *rpl21e* than *rpoF* in the wild type, but only 1.4- and 1.5-fold more in the ioTTS*_rpl21/rpoF_* terminator and ▽*aCPSF1* mutants following a ∼2-fold increase in the *rpoF* transcripts in both mutants (Supplementary Figure S10C). By western blotting, Rpl21e (0.67 pmol/μg) was detected to be 4.3-fold more abundant than RpoF (0.157 pmol/μg) in wild type, but the ratio was reduced to 3-fold in theTTS*_rpl21/rpoF_* mutant due to a 30% increase of RpoF (Supplementary Figure S10D). Thus, ioTTS termination played a role in controlling protein production as well as transcript abundance of the *rpl21e-rpoF* operon.

Collectively, these experiments demonstrated that ioTTSs play important roles in the expression of RP-RNAP operons, especially in controlling the differential transcription and protein production of the two types of genes encoding two essential cellular macromolecular genetic machineries for translation and transcription.

### Functional significance of ioTTS termination in expression of RNAP subunits

**Figure 5.**
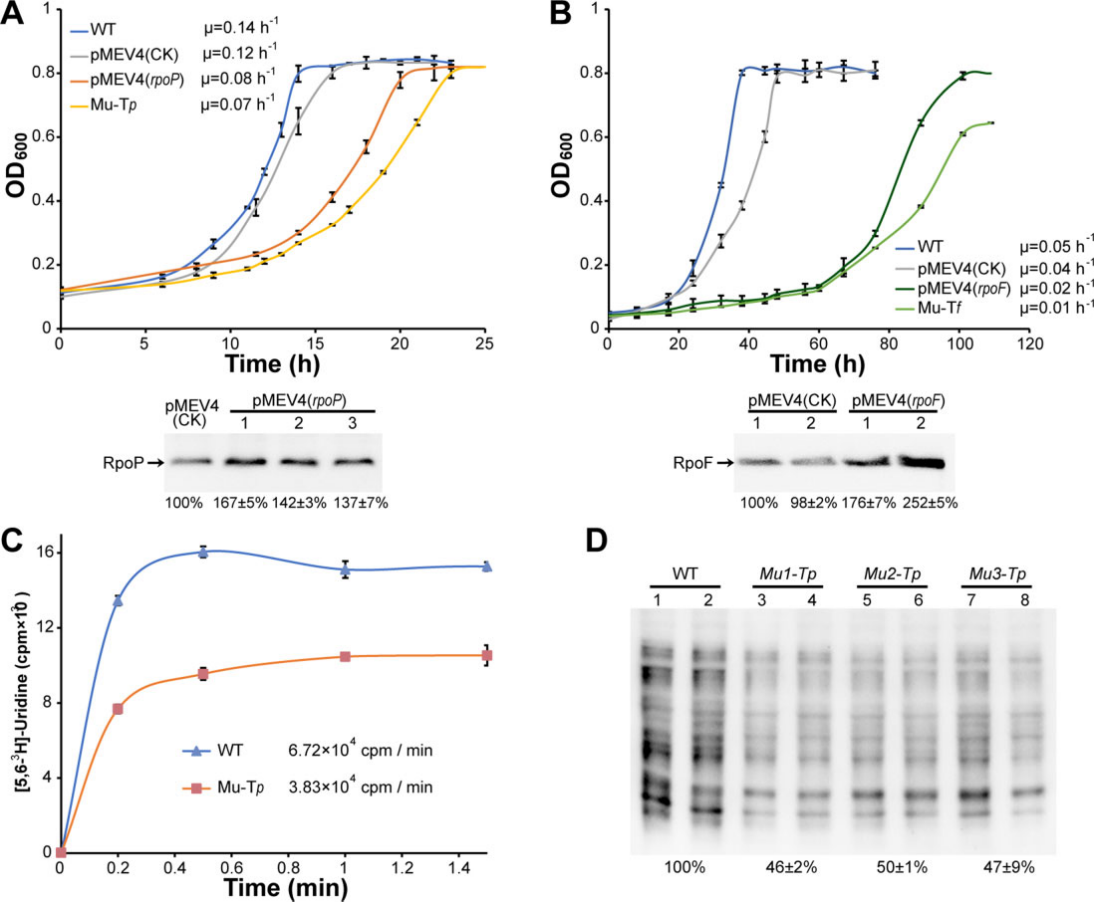
Overexpression of *rpoP* and *rpoF* inhibits growth. RpoP and RpoF were overexpressed either in the shuttle vector pMEV4 or ioTTS*_rpl37Ae/rpoP_* and ioTTS*_rpl21e/rpoF_* terminator mutants. The empty vector pMEV4(CK) served as a control. (**A**, upper) Growth of the wild type (WT), the ioTTS terminator mutant Mu-T*_p_* and the overexpression strain pMEV4(*rpoP)* at 37 ºC. (A, lower) Western blot of the levels of RpoP in the wild type and overexpression strain. (**B**, upper) Growth of the wild type (WT), the ioTTS terminator mutant Mu-T*_f_*, and overexpression strain pMEV4(*rpoF*) at 22 ºC. (B, lower) Western blot of the levels of RpoF in the wild type and overexpression strain. (**C**) Transcription rates of the wild type (WT) and Mu-T*_p_* mutant were compared based on ^3^H-uridine incorporation rate. Numbers in the inset indicate the calculated transcription rates of the WT and mutant by plotting the ^3^H-uridine incorporation amounts vs. the sampling time. Triplicate cultures were assayed, and averages and standard deviations are shown. (**D**) Nascent synthesized protein contents of the wild type (WT) and Mu-T*_p_* mutant were determined based on puromycin integration. The total protein intensity of each lane is quantified by ImageJ, and the averaged intensity of puromycin integration in each of two lanes of wild type and the Mu-T*_p_* mutants are indicated below the gel.

Following the demonstration of the role of ioTTS termination in RNAP gene transcription, the physiological significance of ioTTS termination on RNAP subunit production was investigated. Compared to the growth rate of the wild type of 0.14 h^−1^, the ioTTS*_rpl37/rpoP_* mutant Mu-T*_p_* grew with a half of the rate or 0.07 h^−1^ at 37°C (Figure 5A). While the ioTTS*_rpl21e/rpoF_* terminator mutant Mu-T*_f_* exhibited the same growth rate as the wild type at 37°C, its growth rate was reduced 5-fold to 0.01 h^−1^ at 22°C (Figure 5B). Similarly, *rpoN* and *rpoK* were downstream of the ioTTS*_rps9/rpoN_* and transcribed less than the upstream RP genes (Supplementary Figure S11A and B). A mutation of the ioTTS*_rps9/rpoN_* resulted in a reduced growth at 37°C (Supplementary Figure S11C) as well. To verify that the elevated production of RNAP subunits was responsible for the growth retardation, *rpoP* and *rpoF* were expressed on shuttle vectors. Overexpression was verified by western blotting, and RpoP and RpoF protein productions increased 1.5-fold and 2.1-fold in the strains pMEV4(*rpoP*) and pMEV4(*rpoF*), respectively. Growth of the overexpression strains was also retarded and similar to those of the ioTTS terminator mutants (Figure 5). Thus, an excess of RNAP subunits appeared to cause the growth defects observed in the ioTTS mutants.

RNAP plays a critical role in transcription, and the overall transcription rate of the Mu-T*_p_* was compared with that of the wild type. Based on the ^3^H-uridine incorporation rate, the overall transcription rate of the Mu-T*_p_* mutant was determined to be about half that of the wild type at 22°C (Figure 5C). Because of its rapid growth, it was impractical to measure the rate of ^3^H-uridine incorporation at 37°C. The rate of translation was also impacted in the Mu-T*_p_* mutant. Methanococci are sensitive to puromycin, an aminonucleoside antibiotic and tRNA analogue that covalently binds to the nascent polypeptide chain during active translation. Thus, it can be used to indicate newly synthesized protein (30). Using the anti-puromycin antibody, western blotting determined significantly decreased puromycin-integration in the Mu-T*_p_* mutant compared with that of the wild type (Figure 5D).

In conclusion, ioTTS termination in RP-RNAP operons plays important roles in the normal rates of RNAP production with profound consequences on growth and rates of transcription and translation.

### Importance of intracellular levels of RNAP subunits in RNAP assembly

**Figure 6.**
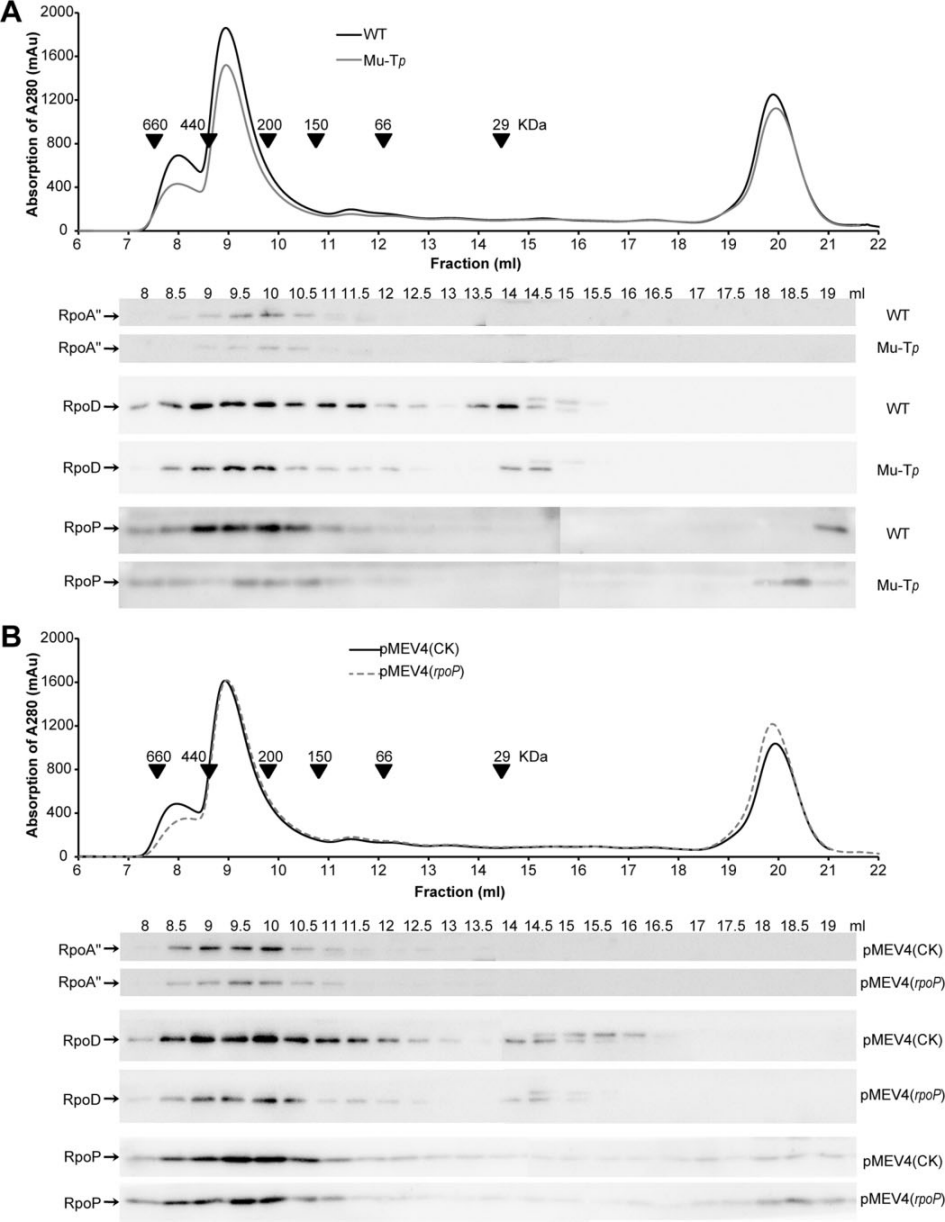
RNAP assembly in the wild type (WT), ioTTS*_rpl37Ae/rpoP_* terminator mutant Mu-T*_p_* (**A**) and RpoP overexpression strain pMEV4(*rpoP*) (**B**). The quantitative western blot assay of size exclusion chromatography (SEC) fractions was used to compare the relative abundances of RNAP complex and free subunits in WT and Mu-Tp mutant. Cell-free extracts of the *M. maripaludis* strains were fractioned by SEC (upper panels). The complete complex, assembly intermediates and free subunits of RNAP in various SEC fractions are quantitatively compared through western blotting (bottom panels) using the polyclonal antibodies against RpoA'', the RNAP catalytic subunit, and RpoD and RpoP, two subunits of RNAP assembly platform. The fractions where the molecular weight markers eluted are indicated. The strain (pMEV4(CK)) carrying the empty overexpression vector served as the wild type control in (B). Triplicate experiments were performed, one representative of which is shown.

To examine why ioTTS termination-modulated RNAP protein expression impacted rates of transcription, the assembly of RNAP was surveyed in the two strains with elevated levels of RpoP, the ioTTS*_rpl37/rpoP_* mutant Mu-T*_p_* and the overexpression strain pMEV4(*rpoP*). RNAP assembly in cell extracts was tested by size exclusion chromatography coupled to western blotting using antibodies against RpoA", RpoP and RpoD. Elution of the fully assembled RNAP with a predicted molecular weight (Mr) of ∼350 kDa was detected in fractions ∼9–10 ml from all tested strains (Figure 6). However, compared with wild type, lower amounts of the fully assembled RNAP complex were detected in both Mu-T*_p_* and pMEV4(*rpoP*) strains. In contrast, both overexpression strains contained ∼2-fold higher levels of free RpoP (eluting at 18–19 ml or ∼6 *K*_d_), lower levels of free RpoD (eluting at 14.5–15 ml or ∼20 *K*_d_) and potential RNAP assembly intermediates, presumably including RpoNPDL (eluting at 13.5–14.5 ml or ∼34 *K*_d_) (Figure 6A and B).

In conclusion, elevation of the levels of a RNAP subunit impaired the assembly of the RNAP complex, and termination at the ioTTS within the RP-RNAP operon was one mechanism to control the production of the appropriate levels of RNAP subunits.

### Wide distribution of the RP-RNAP operons and ioTTS termination among archaea

**Figure 7.**
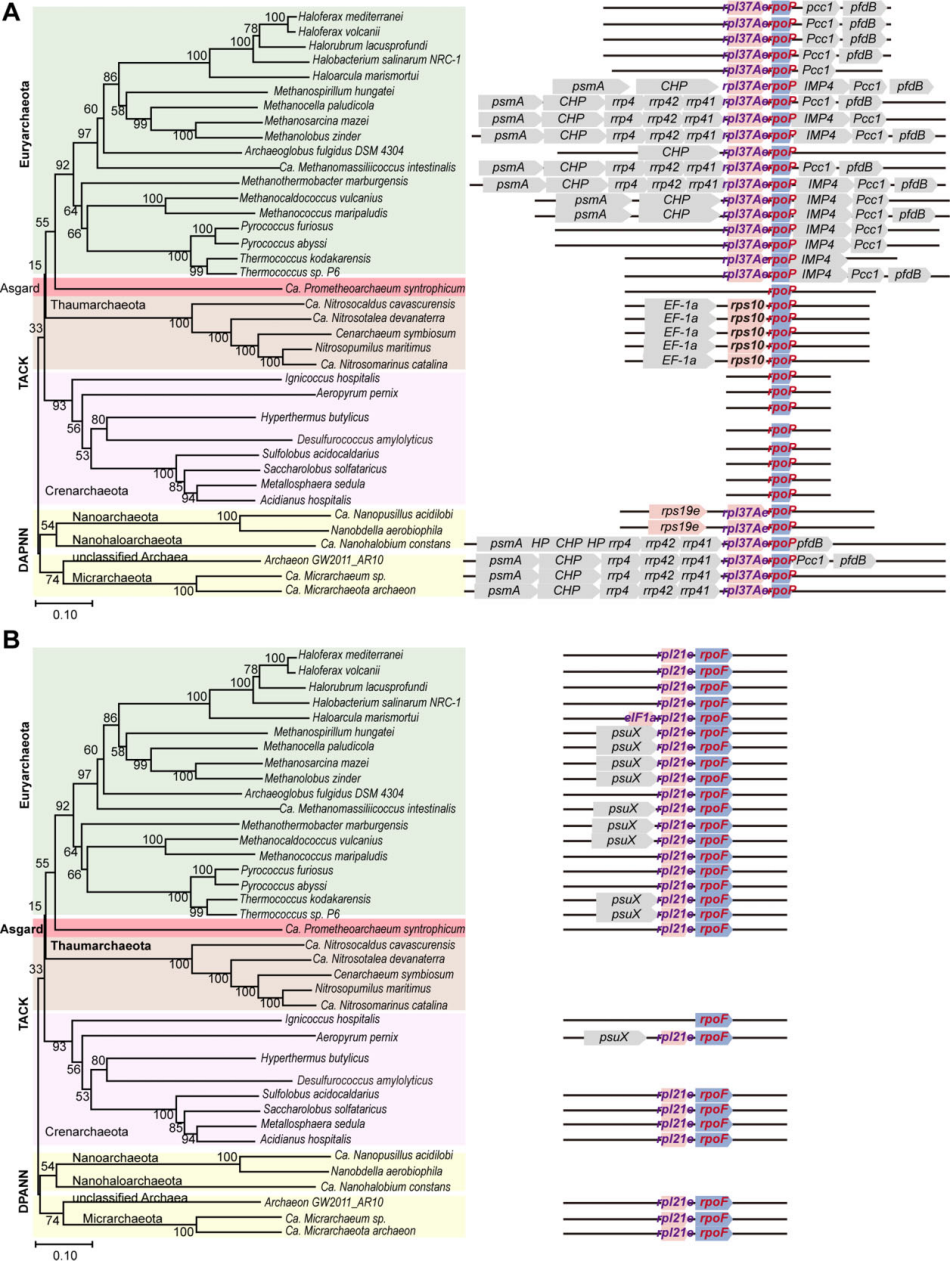
Distribution of the RP-RNAP operons containing *rpoP* (**A**) and *rpoF* (**B**) among the Archaea. (left) Phylogenetic tree of representative archaea constructed by the neighbor-joining of the sequences of concatenation conserved archaeal proteins. Bootstrap values are shown at each node. Bar of 0.10 represents 10% amino acid differences. (right) Conservation of the operons contain *rpoP* and *rpoF*. Only conserved genes are indicated. CHP is conserved hypothetical protein. IMP4 is U3 small nucleolar ribonucleoprotein protein.

Distribution of operons with the *rpl37Ae*-*rpoP* and *rpl21e*-*rpoF* gene organization among diverse archaea was investigated. Using the *M. maripaludis rpoP* as a probe, operons bearing *rpl37Ae*-*rpoP* were found in the Euryarchaeota and superphylum DPANN (Figure 7A) and were often associated with the conserved genes *psmA, IMP4*, *pcc1* and *pfdB*, which encode proteasome alpha subunit, rRNA maturation protein IMP4, KEOPS subunit Pcc1, and prefoldin beta subunit, respectively. Genes encoding exosome subunits Rrp4, Rrp42 and Rrp41 were also found upstream of *rpl37Ae* in many Euryarchaeota and DPANN genomes. Genes encoding EF-1a and Rps10 replaced *rpl37Ae* upstream of *rpoP* in Thaumarchaeaota. Interestingly, stand-alone *rpoP* was found in all Crenarchaeota species, and probably in Ca. *Prometheoarchaeum syntrophicum* of the Asgard group (Figure 7A). Therefore, the gene organization of *rpl37Ae*-*rpoP* was widely but not universally conserved among the archaea.

The operon organization of the RP-RNAP operons across archaeal lineages suggests that an evolutionary transition in genomic arrangements may have occurred. Large operons (up to 10 genes) are found in Euryarchaeota and DPANN species, while shorter operons consisting of 3–4 genes occur in Haloarchaea and Nanoarchaeota. Interestingly, in TACK species, the archaeal clade believed as the closest relatives of eukaryotes (31), the *rpoP* gene is present as stand-alone. This evolutionary trend may reflect a potential link between prokaryotic gene cluster organization and the transition to the single-cistron arrangement seen for eukaryotic genes. Similarly, the gene organization *rpl21e*-*rpoF* was widely distributed but not universal. It was found in the majority of groups that contained *rpoF* homologs, including the Euryarchaeota, the majority of Crenarchaeota and DPANN archaea, and Ca. *P. syntrophicum* (Figure 7B).

Reexamination of the available transcriptomic data of archaea, higher levels of transcription of *rpl37Ae* than that of *rpoP* was found in *Thermococcus kodakarensis*, *T. onnurineus*, *Methanosarcina acetivorans*, and *Methanococcoides burtonii* (Dataset S3). Thus, ioTTS termination may be common within this operon in the Euryarcheaota and awaits further examination in other archaea.

## DISCUSSION

Transcription termination plays critical regulatory roles in gene expression, such as in regulating sRNA function (32–35), riboswitch action (36–38), repressing xenogenic DNA and pervasive transcriptions (39–43) and maintaining chromosome integrity and transcription and translation coupling (38,44,45). However, the process of transcription termination and its significance in regulating physiology remain largely unclear in the third form of life, archaea. Previously, we identified the genome-wide transcription termination sites and demonstrated that transcription termination is required for programming an ordered transcriptome of *M. maripaludis* (22). This study reported that the internal operon (ioTTS) termination tunes differential transcriptions of operon-organized genes. Based on the multi-transcriptomic data, ioTTS termination was found to be common in *M. maripaludis* and have been experimentally verified to be physiological significant. Either destroying the intrinsic ioTTS terminators in selected operons, such as Afu ABC transporter and RP-RNAP operons, or artificially inserting an ioTTS into the operon encoding ATPase complex not only disrupted the inherent stoichiometric expression of the operon genes, but also impaired the physiological functions of the complex (Figures 2–6 and Figs. S7, S9, S10, S11). Therefore, the differential transcription of the operon-organized genes modulated by ioTTS termination is a physiological demand.

Clustering of functionally related genes in operons allows for coregulation and especially even gene expression in prokaryotes. However, achieving precise, and sometimes uneven, stoichiometries in protein complexes requires fine-tuning mechanisms. Translation efficiency, influenced by mRNA secondary structure and codon usage (15,17,18), as well as posttranslational mechanisms including differential protein degradation (46,47), play roles in maintaining protein stoichiometries. Regulatory elements, present in or targeting mRNA 5′ or 3′ untranslated regions (5′UTR or 3′UTR) or regions internal to ORFs, and RNA polymerase pausing also contribute to fine-tune gene expression (16,20,48–50). In this study, we experimentally demonstrate the physiological significance of ioTTS termination as an additional effective regulatory mode that fine-tunes the stoichiometric expression of operon genes. Unlike the transcription polarity effect observed in bacteria, which arises from the dissociation of transcription and translation when translation is halted, usually at nonsense mutations or ribosome pausing, and resulting in premature transcription termination so affecting the expression of downstream genes (51–55), ioTTS termination occurs under normal physiological conditions. Through terminating partial transcriptions from the operon promoter and allowing part of transcription readthrough, ioTTS termination controls transcription of the genes downstream of ioTTSs to be lower than that of the upstream ones and the subsequent protein production, assembly of protein complexes, and growth. ioTTS termination can not only explain 30–80% transcription termination efficiency (TTE) determined in many of TTSs in *M. maripaludis* (21,22) but also can echo the finding that extensive transcription read-through was found at over 40% of TTSs in *E. coli* and several other bacteria (16,19,20), in which similar transcription differences occurred at genes flanking the ioTTS within operons. Importantly, ioTTS termination was found to be a novel regulatory mechanism in controlling the precise expression of the RNAP subunits in the RP-RNAP operons. Mutation of the ioTSS terminators in the RP-RNAP operons, *rpl37Ae-rpoP, rpl21e-rpoF, and rps9-rpoNK*, elevated transcription of the ioTSS downstream *rpo* genes, but instead reduced the cellular RNAP complex contents, and transcription and translation velocities (Figures 4, 5, (6, S9, S10, and S11), and the consequent growth of *M. maripaludis*. Proper cellular contents of RNAP subunits appear to be required in assembling the complete RNAP complex, and termination at ioTTSs appear to be one of the control strategies. The *in vivo* assembly process of archaeal RNAP complex and the regulation of its expression and assembly all remain unknown. The archaeal RNAP assembly process was predicted based on *in vitro* reconstitution experiments (56). In which, the subunits RpoD, RpoL, RpoN, and RpoP are first fit together as an assembling platform (57,58), and on it the catalytic subunits RpoB′, RpoB″, RpoA′ and RpoA″ followed by RpoH and RpoK are assembled. Finally, the accessary subunits RpoE and RpoF are recruited (58,59). In the current study, through antibodies targeting the catalytic and assembly platform subunits, RpoA″ and RpoD/RpoP subunits respectively, we found that elevation of the RNAP subunit RpoP in both the ioTTS*_rpl37Ae-rpoP_* terminator mutation (Mu-T*p*) and the pMEV4-*rpoP* overexpression strain, instead decreased the cellular content of the complete RNAP complex and a potential assembly intermediate (Figure 7). This indicates that appropriate contents of one or more RNAP subunits or certain cellular ratio among the subunits is required for the effective assembly of the archaeal RNAP complex. Supportively, a precise content of Rpb10, a eukaryotic RNAP subunit, is required in eukaryotic RNAP I and RNAP III assembly as indicated by genetic studies (60–62). Rpb10 is the homolog of archaeal RpoN, and functions as the RNAP assembly platform by serving as a structural adaptor to fit the β- and α-like catalytic Rpo2 (RpoB) and Rpo3 (RpoL) subunits (63,64). Recent studies indicated that the cellular concentrations of Rpb10 are precisely controlled through a reciprocal regulation of an RNA binding protein Rbs1 and a Upf1 helicase (60–62). Analogously, the precise contents of RpoP and RpoN, the platform subunits, could also be key to the archaeal RNAP assembly, and ioTTS termination can be one of the control strategies.

ioTTS termination in coordinating expression of the archaeal and bacterial RP-RNAP operons can exert an important role in physiology, representing a novel regulatory mode that not only ensures the proper levels of the suite of subunits that assemble into the complete RNAP complex but also in coordinating the production of the systems to couple translation and transcription. Coupled transcription and translation in prokaryotes is usually achieved by ribosomes loaded on the transcripts being transcribed, and the leading ribosome even binding to the processive RNAP (65). In active *E. coli*, ribosomal proteins account for up to 40% of the total cellular proteins, and multiple ribosomes can synchronically translate one transcript (66,67). Thus, it reasonably predicts that higher abundances of ribosomal than RNAP proteins are in cells, and this was confirmed in *M. maripaludis* where the ratios of Rpl37Ae to RpoP and Rpl21e to RpoF are determined to be 14.3:1 and 4.3:1, respectively. While, changing the ratios by mutation of ioTTS terminators reduced transcription and translation velocities and also growth of *M. maripaludis* (Figures 4, 5, S10 and S11). This suggests that the appropriate proportion between the subunits of the translational and transcriptional macromolecules should be the physiological demands and are controlled at least in part by termination at ioTTSs.

Given the wide distribution of the RP-RNAP operons in archaea and presumably differential transcription of RNAP and RP subunit genes in the operons of some archaeal species (68–71) and dataset S3, the ioTTS termination regulation might be a common strategy employed by archaea. Therefore, the operon organization of RP and RNAP subunits, the two macromolecular machineries that are involved in translation and transcription, could be evolutionarily advantageous to ensure the optimal coupling of the two processes, and ioTTS termination could be a widely employed regulatory mode for controlling the required levels of them in cells.

In conclusion, this work reports that ioTTS termination functions as a new regulatory mode in coordinating differential expression of genes in archaeal operons that encode protein complexes consisting of uneven stoichiometry of subunits or involving in different biological processes. The biological significance of this type of coordinated differential expression is particularly illustrated by the RP-RNAP operons, where disruption of this coordination reduced RNAP complex content and as a consequence transcription, translation, and growth. The ioTTS termination not only demonstrates the complexity of prokaryotic transcription regulation mechanisms but illustrates a novel mode of controlling the precise RNAP subunit content. Thus, ioTTS termination-controlled RP-RNAP operon expression is likely widely employed in prokaryotes.

## Supplementary Material

gkad575_Supplemental_Files

## Data Availability

The PacBio-seq data have been deposited at the NCBI SRA Submission BioProject ID PRJNA933779 [https://www.ncbi.nlm.nih.gov/bioproject/PRJNA933779]. This project contains two biosamples. Sequencing data are available on SRA: SRR23410387, SRR23410387.
