# Supplementary material for: Internal transcription termination widely regulates differential expression of operon-organized genes including ribosomal protein and RNA polymerase genes in an archaeon
Source: Nucleic Acids Res. 2023 Jul 13;51(15):7851–67. doi: 10.1093/nar/gkad575 (PMC10450193; doi:10.1093/nar/gkad575)
Supplement: gkad575_Supplemental_Files [file gkad575_supplemental_files.zip › LetPub - Certificate 2023.pdf]

Certificate of English Language Editing

Manuscript Title:

A novel regulatory mode in physiology demanded uneven transcription of operon-organized genes

Date of Revision:

March 9, 2023

Abstract:
Genes organized within operons in prokaryotes benefit from a coordinative even expression; however, some operon genes involved in different biological processes or uneven stoichiometries are unevenly expressed, but the regulatory modes and physiological significances remain largely elusive. This study integrated transcriptomic PacBio-seq, dRNA-seq, Term-seq, and Illumina-seq approaches and identified internal transcription termination sites (ioTTSs) within 38% of the operons of *Methanococcus maripaludis*, an archaeal representative. There were higher transcript and protein abundances in the ioTTS upstream than in the downstream genes within many operons, and transcription termination at ioTTS was demonstrated to coordinate the uneven expression levels of the operon genes via the ioTTS terminator mutations. In particular, mutation of the ioTSS terminators in ribosomal protein (RP)-RNA polymerase (RNAP) operons elevated the expression of the ioTSS downstream RNAP subunits, but decreased the assembled RNAP complex contents, transcription and translation velocities, and growth of *M. maripaludis*...

This document certifies that the manuscript listed above was copy edited for English language by LetPub, with regard to grammar, punctuation, spelling, and clarity. All of our language editors are native English speakers with long-term experience in editing scientific and technical manuscripts. We are committed to leveling the playing field for researchers whose native language is not English.

- Documents receiving this certification should be regarded as having undergone professional editorial revision for English language before submission. However, the authors may accept or reject LetPub's suggestions and changes at their own discretion and LetPub does not have editorial control over the submitted documents.
- The language quality of the submitted document is the sole responsibility of the submitting authors subject to those authors' adherence to LetPub's revisions and instruction. LetPub's provision of service does not constitute a guarantee or endorsement of the authors' work herein.
- Neither the research content nor the authors' intended meaning were altered in any way during the editing process.
- If you have any questions or concerns about this edited document, please contact us at support@letpub.com

LetPub
Your Manuscript Editor

LetPub is an author service brand owned and operated by Accdon LLC. Headquartered in the Boston area, we are a full-spectrum author services company with a large team of US-based certified language and scientific editors, ISO 17001 accredited translators, and professional scientific illustrators and animators. We advocate ethical publication practices and are an official member of the Committee on Publication Ethics (COPE).

For more information about our company, services, and partnership programs, please visit www.letpub.com.

© 2023 Accdon, LLC. All Rights Reserved.   Tel: 1-781-202-9968   Email: info@accdon.com   Address: 400 Fifth Ave, Suite 530, Waltham, MA 02451, United States
