# Supplementary material for: Internal transcription termination widely regulates differential expression of operon-organized genes including ribosomal protein and RNA polymerase genes in an archaeon
Source: Nucleic Acids Res. 2023 Jul 13;51(15):7851–67. doi: 10.1093/nar/gkad575 (PMC10450193; doi:10.1093/nar/gkad575)
Supplement: gkad575_Supplemental_Files [file gkad575_supplemental_files.zip › Supplementary material-R1-Clean.pdf]

Supplementary Information for

Internal transcription termination widely regulates differential expression of the operon-organized genes including ribosomal protein and RNA polymerase genes in an archaeon

Wenting Zhang[1,2], Derong Ren[1,2], Zhihua Li[1,2], Lei Yue[1,2], William B. Whitman[3], Xiuzhu Dong[1,2,*], Jie Li[1, *]

1, State Key Laboratory of Microbial Resources, Institute of Microbiology, Chinese Academy of Sciences, Beijing 100101, PR China

2, University of Chinese Academy of Sciences, No.19A Yuquan Road, Shijingshan District, Beijing 100049, China

3, Department of Microbiology, University of Georgia, Athens, Georgia, 30602, United States of America

*, Correspondence to: Jie Li, No.1 Beichen West Road, Beijing 100101. Tel. 86-10-6480 7567. Email: lijie824@im.ac.cn; Xiuzhu Dong, No.1 Beichen West Road, Beijing 100101. Tel. 86-10-6480 7413, Email: dongxz@im.ac.cn.

This file contains:

Supplementary Figure S1 to S11.

Supplementary Tables S1 to S4.

Caption for Supplementary Dataset S1: Defined operons of *M. maripaludis* based on PacBio-seq combined with data of dRNA-seq, Term-seq and Illumina-seq.

Caption for Supplementary Dataset S2: Transcription profiles of the operons containing ioTTS. Transcription abundances, ioTTS differential expression ratios (TDER), transcription termination efficacy (TTE), and aCPSF1 dependency of these operons are shown.

Caption for Supplementary Dataset S3: Transcription abundances of the *rpl37Ae_rpoP* genes in different organisms.

Supplementary Figures and Figure legends

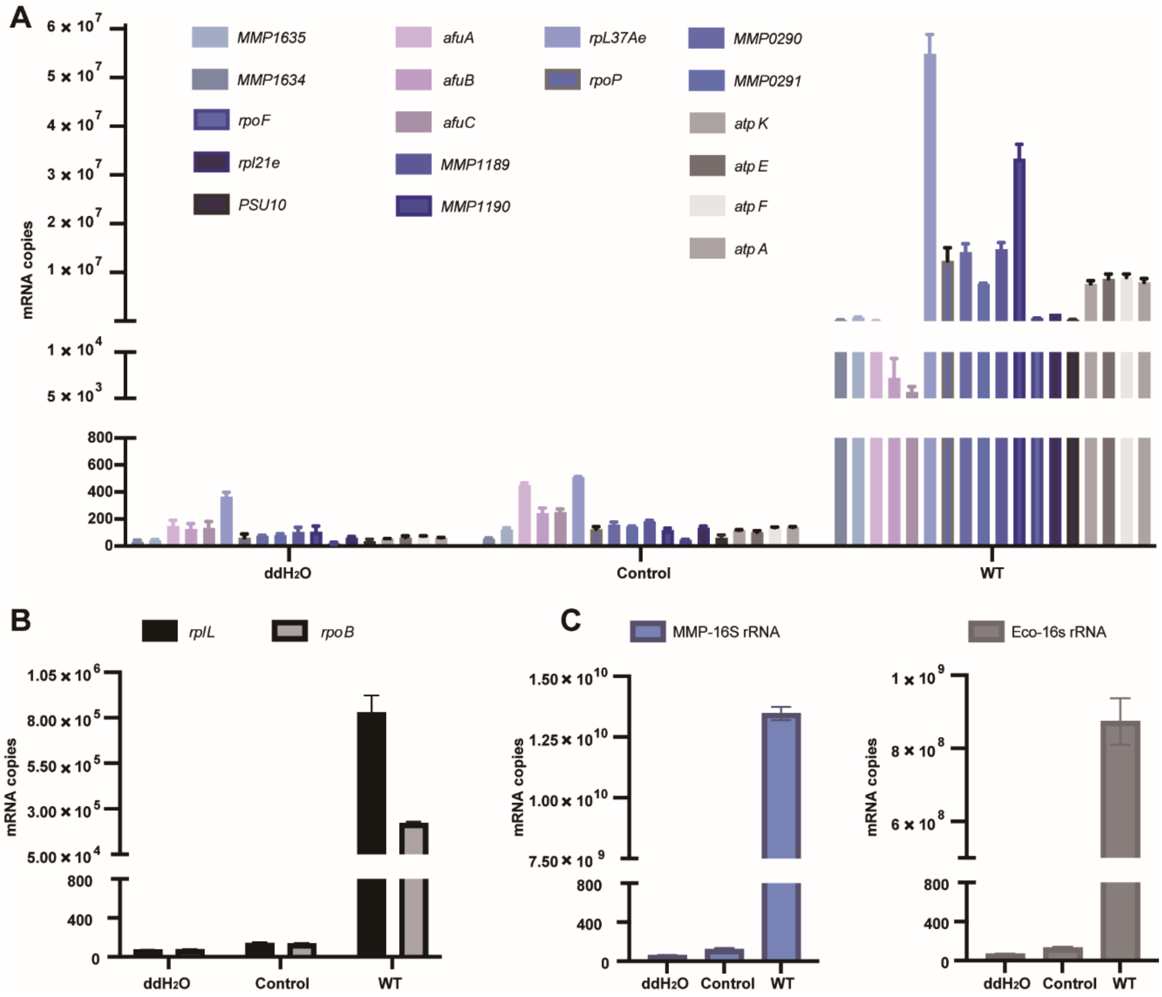

Fig. S1. RT-qPCR control for DNA-free RNA samples to ensure the absence of DNA contamination in the RNA samples. (A) The mRNA copies of selected genes in ddH2O, without RT reaction (control) and the wild-type *M. maripaludis* strain RNAs (WT) were determined parallely by RT-qPCR assays. (B) Similar as in (B), the mRNA copies for rplL and rpoB in ddH_2O, control and wild-type *E. coli* RNA (WT) were determined parallely by RT-qPCR assays. (C) The mRNA copies for 16S rRNAs of *M. maripaludis* and *E. coli* in ddH2O, control and separate RNA samples were determined parallely by RT-qPCR assays. These controls were critical to ensuring the accuracy of our subsequent analyses.

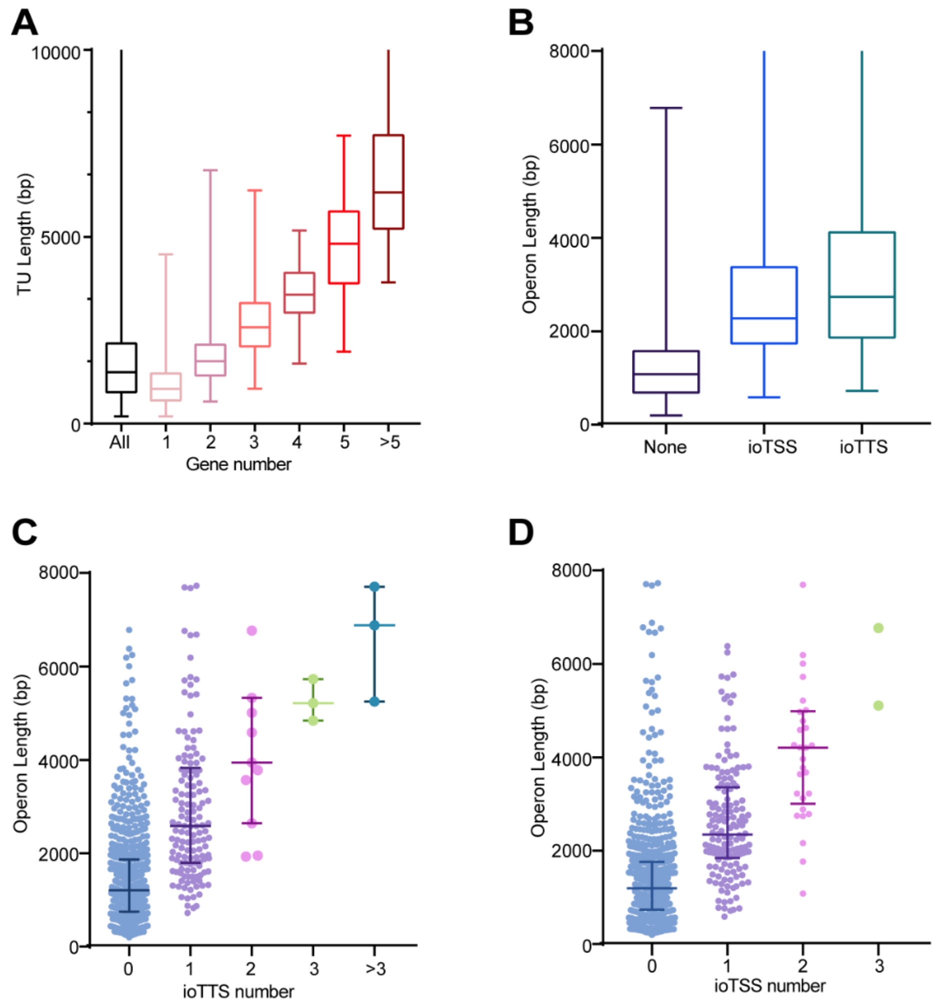

Fig. S2. Summary of operon organization in *M. maripaludis.* (A) Box plot analysis of the correlation of the operon length with the number of genes, (B) Lengths of type III and IV operons with internal transcription-start sites (ioTSSs) and type II and IV operons with internal termination sites (ioTTSs) or type I operons without either one (none), (C) Length of multigene operons depending upon the number of ioTTSs. (D) Length of multigene operons depending upon the number of ioTSSs. The boxes denote the lengths of 50% of the operons, the lines inside the boxes are the operon length medians, and the vertical lines outside the boxes denote the ranges.

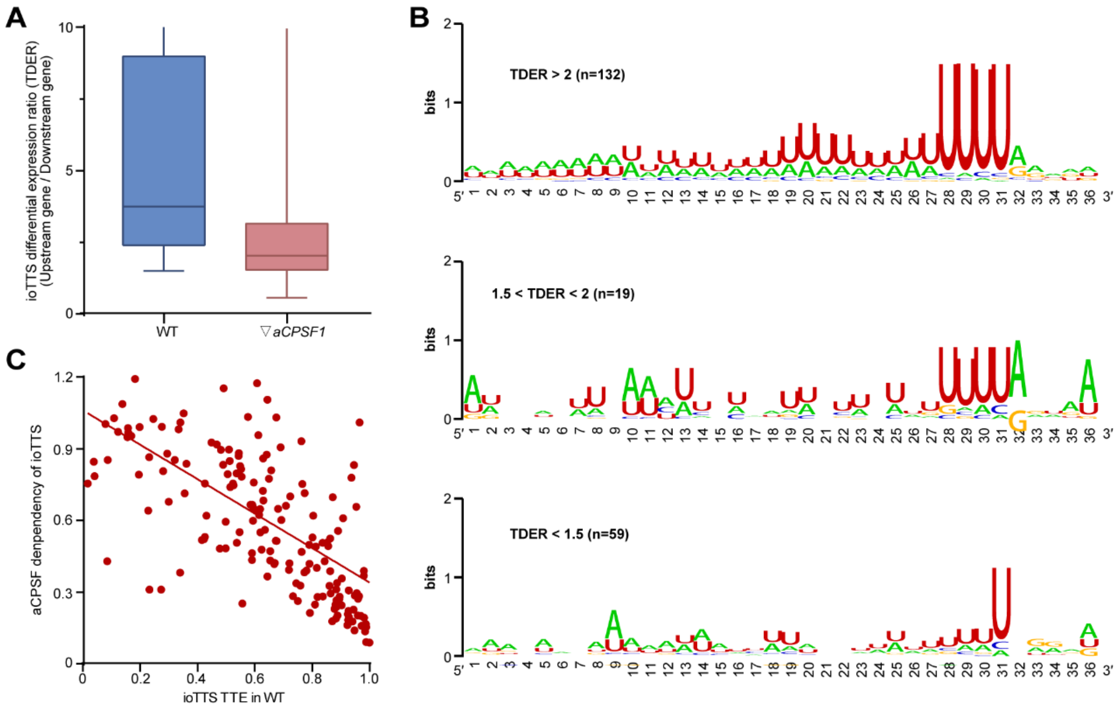

Fig. S3. Role of ioTTSs on flanking gene expression and the importance of the termination factor aCPSF1 and the ioTTS terminator U-tract. (A) Box plots of the TDERs for the wild-type (WT) and aCPSF1-depletion mutant (∇*aCPSF1*). The boxes denote the TDERs of 50% of the ioTTSs, the lines inside the boxes are the median TDERs, and the vertical lines outside the boxes denote the ranges. (B) ioTTS terminator motifs of the operons having TDER of >2, >1.5 and <2, and <1.5. The number of operons having the corresponding TDERs listed in Dataset S2 are inside the parentheses. (C) Correlation of ioTTS termination efficacy (TTE) with aCPSF1- dependency of the TDERs. The aCPSF1 dependency and TTE were calculated as described in the methods and Dataset S2.

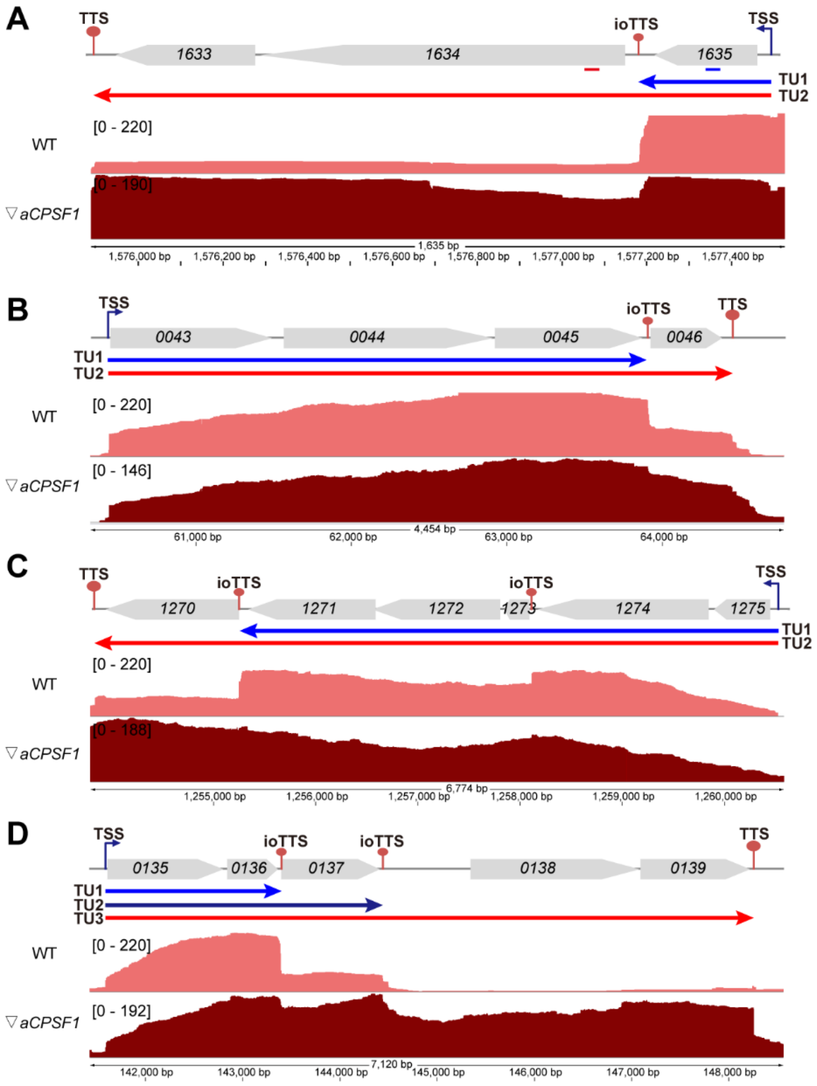

Fig. S4. **Representative PacBio-seq profiles show differential transcription of the ioTTS flanking genes in wild-type (WT) and aCPSF1-depletion mutant (∇*aCPSF1*).** Numbers inside bullets indicate the *M. maripludis* genes, and numbers inside brackets are the number of PacBio-seq reads. TU, transcription unit; TSS and TTS, transcriptional start and termination sites of an operon, respectively; ioTSS and ioTTS, intergenic transcriptional start and termination sites within an operon, respectively.

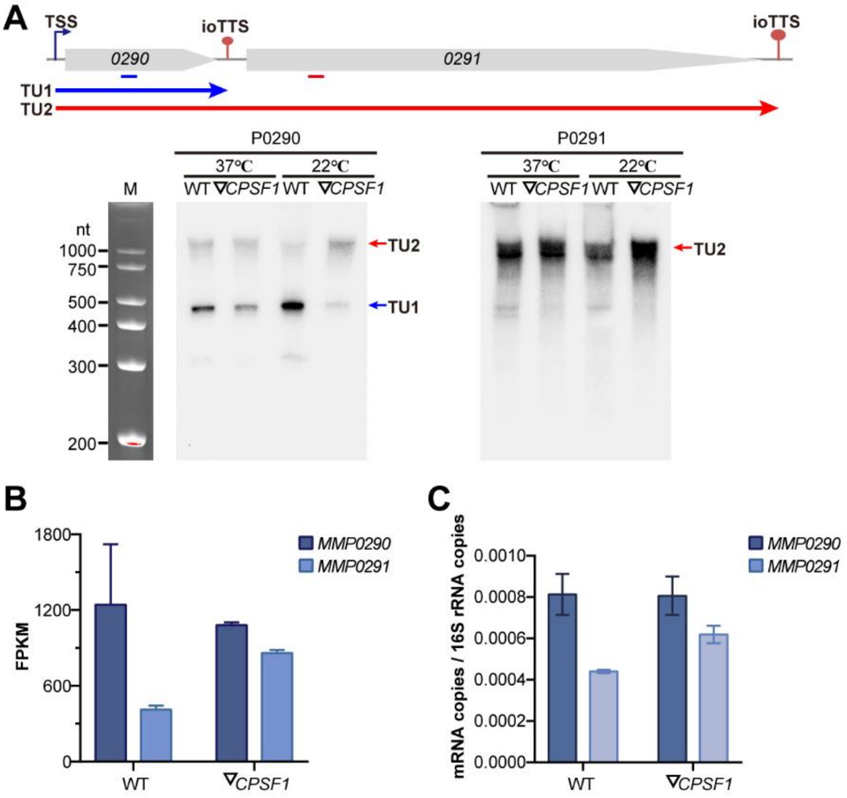

Fig. S5. **Experimental verification of differential transcription at the ioTTS in the *MMP0290-0291* operon**. (A upper) Schematic of the gene organization, transcript unit (TU), TSS, ioTTS and TSS predicted in operon *MMP0290-0291* based on PacBio-seq combined with dRNA-seq and Term-seq. (A lower) Northern blot assays of the transcription units in the wild-type (WT) and aCPSF1 depletion mutant ∇*aCPSF1* growing at 37°C and 22°C using probes targeting the genes as indicated in the upper panel of A. (B) Transcript abundances of the genes flanking the ioTTS in the wild-type and ∇aCPSF1 mutant following growth at 22°C were determined by Illumina-seq. (C) Transcript abundance determined by qRT-PCR. For B and C, experiments were performed on triplicate cultures, and the averages and standard deviations are shown.

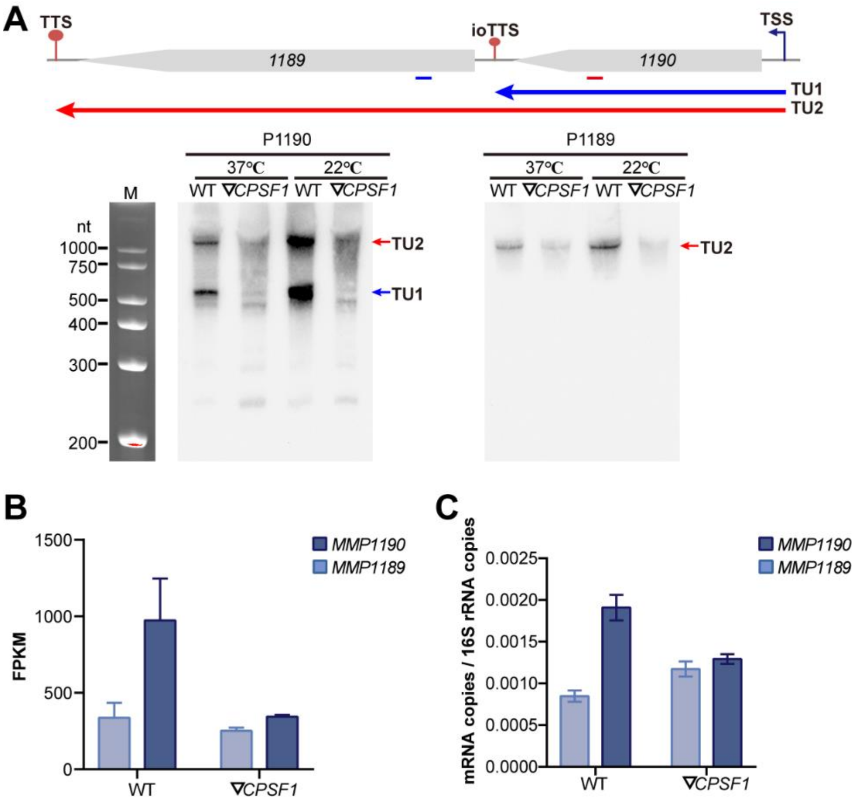

Fig. S6. **Experimental verification of differential transcription at the ioTTS in the of the *MMP1190-1189* operon**. (A upper) Schematic of the gene organization, transcript unit (TU), TSS, ioTTS and TSS predicted in operon *MMP1190-1189* based on PacBio-seq combined with dRNA-seq and Term-seq. (A lower) Northern blot assay of the transcription units in the wild-type (WT) and aCPSF1 depletion mutant ∇*aCPSF1* growing at 37℃ and 22℃ using probes targeting the genes as indicated in the upper panel of A. (B) Transcript abundances following growth at 22℃ of the genes flanking the in the wild-type and ∇aCPSF1 strains were determined by Illumina-seq. (C) Transcript abundance determined by qRT-PCR. For B and C, experiments were performed on triplicate cultures, and the averages and standard deviations are shown.

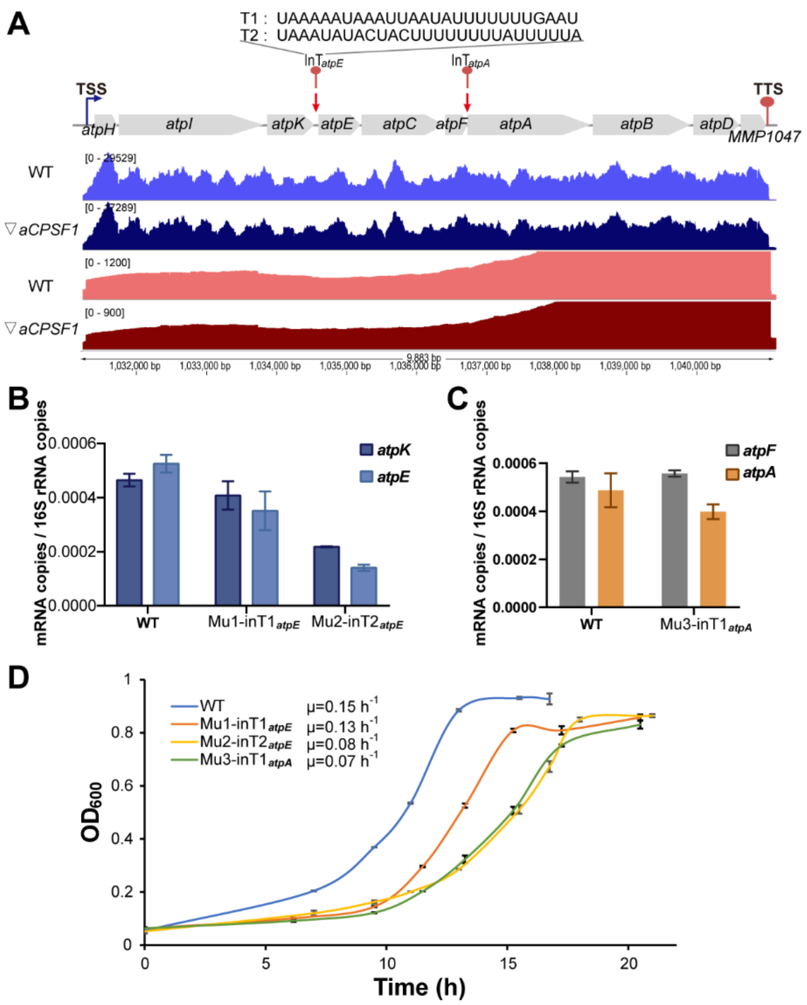

Fig. S7. **Effect of insertion of ioTTSs in ATP synthase operon.** (A) Illumina-seq (blue, dark blue) and PacBio-seq (red, brown) maps of the transcription profiles of the 10-gene operon encoding ATP synthase complex in the wild type (WT) and ▽*aCPSF1* mutant. Notation of TSS, TTS, and numbers in bracket and at bottom are all shown as in Figure 1A. Two terminators, T1 and T2 (sequences at the top of A), were inserted into the IGRs between *atpK* and *atpE* and *atpF* and *atpA* to obtain mutants Mu-inT1$_{atpE}$, Mu-inT2$_{atpE}$, and Mu-inT1$_{atpA}$, respectively. (B) qRT-PCR quantification of the transcript abundances of *atpK* and *atpE* in the wild type and mutants Mu-inT1$_{atpE}$, Mu-inT2$_{atpE}$ and (C) *atpF* and *atpA* in the wild type and mutant Mu-inT3$_{atpA}$. (D) Growth of the wild type and three mutants Mu-inT1$_{atpE}$, Mu-inT2$_{atpE}$, and Mu-inT1$_{atpA}$.

Triplicate cultures were assayed, and the averages and standard deviations are shown. Cultures were grown at 22℃ in part A and 37℃ in parts B-D.

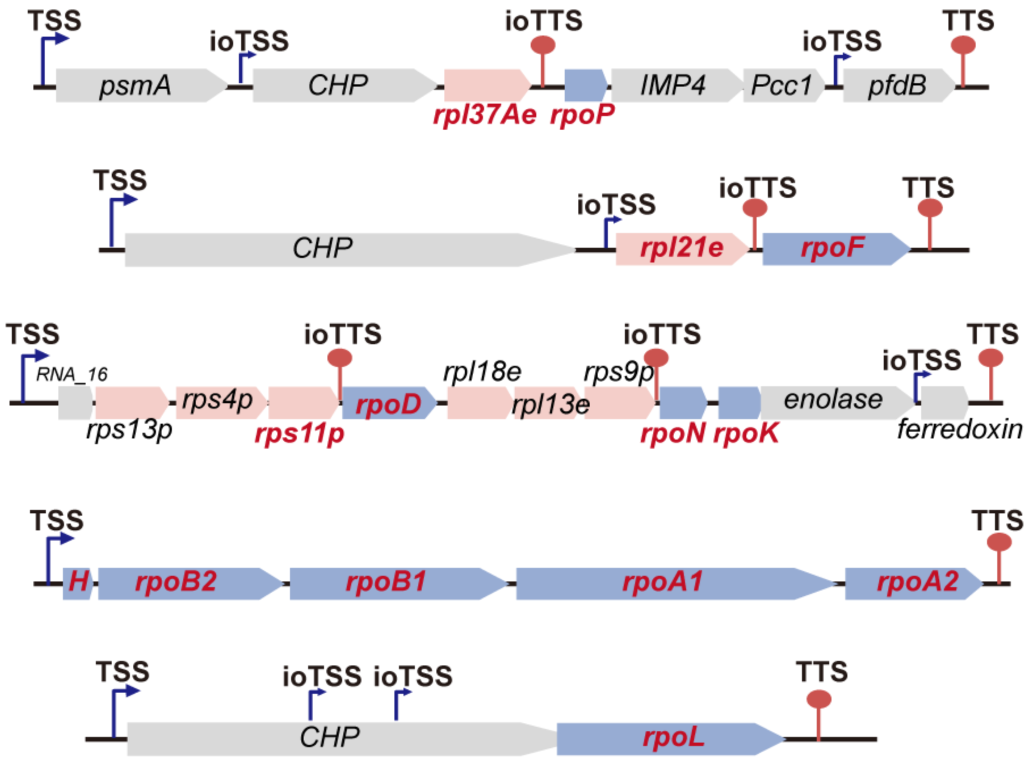

Fig. S8. Map of the operons encoding ribosomal proteins (*rp,* pink bullets) and RNA polymerase subunits (*rpo,* blue bullets) in *M. maripaludis*. Genes encoding other proteins are grey bullets. Operon organization was defined on the basis of PacBio-, Illumina-, dRNA- and Term-sequenced transcriptomic data. TSS, operon transcription start sites; TTS, operon transcription termination sites; ioTSS and ioTTS, operon internal transcription start and termination sites, respectively. CHP, conserved conserved hypothetical protein.

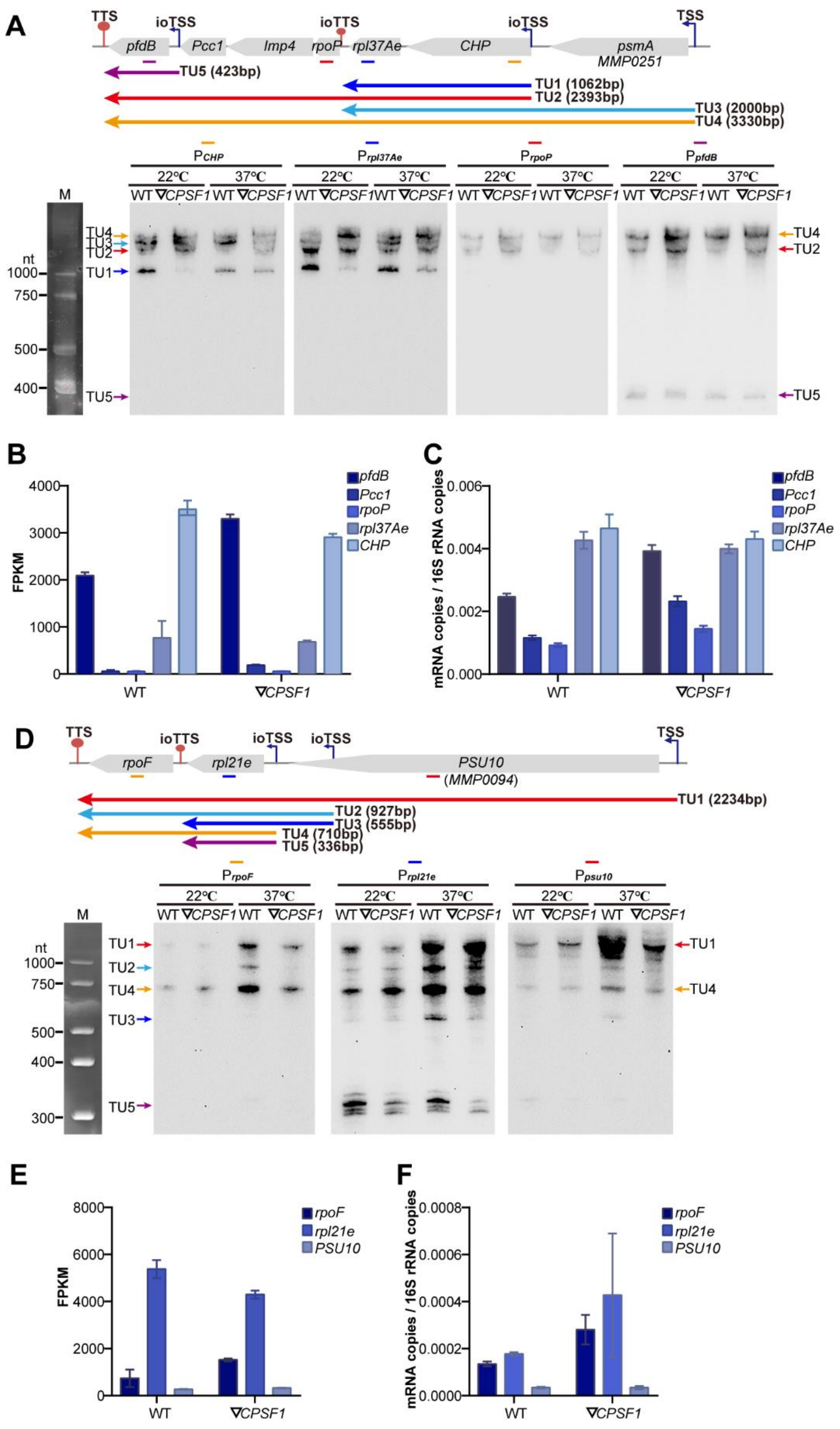

Fig. S9. **Importance of ioTTSs in differential expression in the *rpl37Ae-rpoP* and *rpl21e-rpoF* operons.** (A, upper) Map of transcription in the *rpl37Ae-rpoP* operon. The transcription units based on PacBio- and Illumina-sequencing data are shown as colored

arrows. TSS, ioTSS, ioTTS, and TTS are the same as in Figure 1A. (A, lower) Northern blot assays of the transcription profiles of the *rpl37Ae-rpoP* operon in the wild type and ▽*aCPSF1* mutant grown at 37℃ and 22℃. The probes used are indicated by the colored bars in the upper portion of A, and their sequences are given in Supplementary Table S4. The ssRNA markers (M) serve as a reference. (B) Illumina sequenced transcript abundances (FPKM) of the genes encoded in *rpl37Ae-rpoP* in wild type and ▽*aCPSF1* mutant at 22℃. (C) qRT-PCR quantification of transcript abundances of the genes in the *rpl37Ae-rpoP* operon in wild type and ▽*aCPSF1* mutant. Primers used are listed in Supplementary Table S4. Triplicate cultures were assayed, and the averages and standard deviations are shown. (D, upper) Map of transcription in the *rpl21e-rpoF* operon. The transcription units based on PacBio- and Illumina-sequencing data are shown as colored arrows. (D, lower) Northern blot assays of the transcription profiles of the *rpl21e-rpoF* in the wild type and ▽*aCPSF1* mutant grown at 37℃ and 22℃. Otherwise as in A. (E) Illumina sequenced transcript abundances (FPKM) of the genes encoded in the *rpl21e-rpoF* operon in wild type and ▽*aCPSF1* mutant at 22℃. (F) qRT-PCR quantification of transcript abundances of the genes in the *rpl21e-rpoF* operons in wild type and ▽*aCPSF1* mutant. Otherwise as in C.

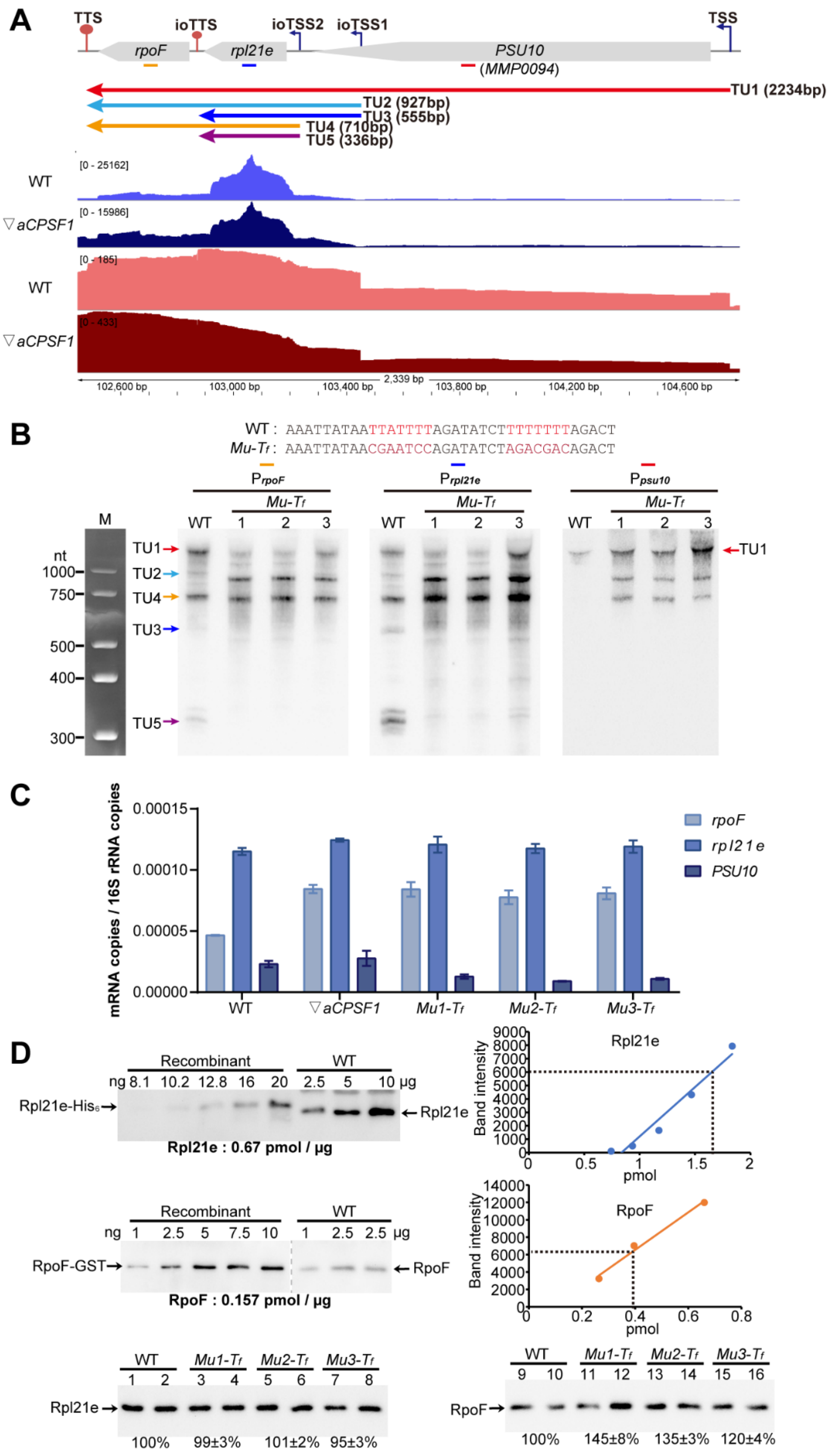

Fig. S10. ioTTS termination regulates expression in the *rpl21e-rpoF* operon. (A) Map of transcription in the operon determined by Illumina-seq (blue, dark blue) and

PacBio-seq (red, dark red) in the wild-type (WT) and ∇*aCPSF1* mutant. The transcription units predicted by sequencing are shown as colored arrows. TSS, ioTSS, ioTTS, TTS, and numbers in brackets and at bottom are the same as in Figure 1A. (B) The ioTTS$_{rpl21e/rpoF}$ terminator (T_f) was mutated by replacing the T-rich tract of the wild type with the sequence Mu-T_f shown at the top of B. Three DNA probes, whose locations are indicated with colored bars in A and whose sequences are shown in Supplementary Table S4, were used in northern blot assays of the operon transcription profiles in the wild type and three replicate colonies of the mutant Mu-T_f. By reference to the ssRNA marker (M), transcript units (TUs) illustrated with the colored arrows in A were determined. (C) qRT-PCR quantification of the transcript abundances of *rpl21e, rpoF* and *psu10* in the wild type (WT), the ∇*aCPSF1* mutant, and three replicate colonies of the mutant Mu-T_f. Triplicate cultures were assayed, and the averages and standard deviations are shown. (D) Rpl21e and RpoF protein contents in wild type and Mu-T_f mutants were quantified by western blotting using anti-Rpl21e-his$_6$ and anti-RpoF-GST polyclonal antibodies, respectively. Recombinant proteins of Rpl21e-his$_6$ and RpoF-GST purified from *E. coli* were used for construction of the standard curve. Western blots are shown in the top two left panels and the standard curves in the top two right panels. Percentages of Rpl21e and RpoF abundances in three colonies of Mu-T_f (bottom panel, lanes 3-8 and 11-16) compared to that in the wild type (bottom panel, lanes 1-2 and 9-10). Averages and standard deviations are shown for each pair.

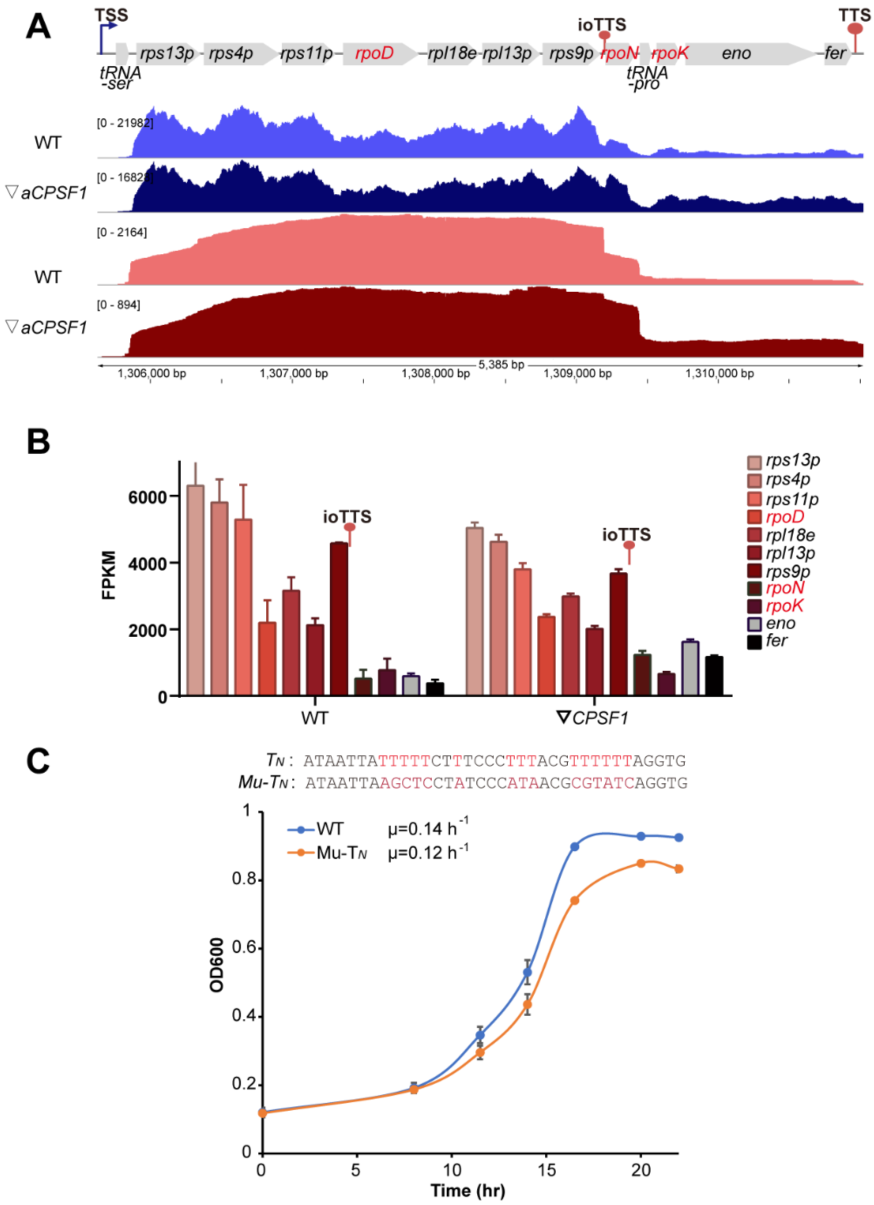

Fig. S11. ioTTS termination regulates differential expression in the *rps9-rpoNK* operon. (A) Map of transcription in the operon determined by Illumina-seq (blue, dark blue) and PacBio-seq (red, dark red) in the wild-type (WT) and ▽*aCPSF1* mutant. TSS, ioTSS, ioTTS, TTS, and numbers in brackets and at bottom are the same as in Figure 1A. (B) Transcript abundances (FPKM) of the genes in wild tupe (WT) and ▽*aCPSF1* mutant determined by Illumina-seq. (C) The ioTTS$_{rps9\text{-}rpoNK}$ terminator (T_N) within the *rps9-rpoNK* operon was mutated by replacing the T-rich tracts shown at the top to obtain the terminator mutant, Mu-T_N. Growth of the wild type and *Mu-*T_N mutant were

determined at 37°C. Triplicate cultures were assayed, and the averages and standard deviations are shown.

Table S1. Strains and plasmids used in this study

**Strains and plasmid**	**Characteristics and descriptions**	**Reference or sources**
**Strains**		
E. coli DH5α	F-φ80d *lac*ZΔM15 Δ(*lac*ZYA-*arg* F) U169 *end*A1 *rec*A1 *hsd*R17(rk-,mk+) *sup*E44λ- *thi* -1 *gyr*A96 *rel*A1 *pho*A	TransGen, Beijing
E. coli BL21(DE3)pLysS	F- *omp*T *hsd*S(rB-mB-) *gal dcm*(DE3)pLysS Camr	TransGen, Beijing
E. coli K-12 MG1655	Wild-type *E. coli*	CGSC 6300
MG1655-*pEcCas*	Wild-type *E. coli* carrying pEcCas	[1]
E. coli M-T$_{rplL\text{-}rpoBC}$	MG1655 *E. coli* with multiple mutagenesis of terminator of b3986	This study
M. maripaludis S0001	Wild-type *M. maripaludi* with Δhpt + ORF1 of pAW42	[2]
Mmp-M-T$_{afuABC}$	S0001 with multiple mutagenesis of terminator of MMP0108	This study
Mmp-M-T$_{rpl37\text{-}rpoP}$	S0001 with multiple mutagenesis of terminator of MMP0249	This study
Mmp-M-T$_{rpl21\text{-}rpoF}$	S0001 with multiple mutagenesis of terminator of MMP0093	This study
Mmp-pMEV4-P0386-mCherry	pMEV4-mCherry, Pur^R,<br>*S0001* with mCherry complement	This study
Mmp-pMEV4-P0386-rpoP	pMEV4-rpoP, Pur^R,<br>*S0001* with MMP0248 complement	This study
Mmp-pMEV4-P0386-rpoF	pMEV4-rpoF, Pur^R,<br>*S0001* with MMP0092 complement	This study
**Plasmids**		
pMD19-T	Amp^R	Takara, Japan
pMD19-T-b3986-donor	pMD19-T with *donor* (b3986-b3987 with point mutagenesis of T$_{b3986}$ sequence)	This study
pEcCas	Kana^R	addgene No. 73227
pEcgRNA	Spec^R	addgene No. 166581
pEcgRNA-N20-b3986	pEcgRNA, ccdB::N20-b3986, targeting the rplL-rpoBC terminator, Spec^R	this study
pMEV4-Cas9-△*Mmp1197* (pMEV403)	pMEV4 containing donor, Cas9 ORF and a guide sequence targeting to *Mmp1197,* Amp^{R,} Pur^R	[3]
pMEV4-Cas9-M-T$_{afuABC}$-sg	pMEV4- *Cas9* with sg targeting T$_{0249}$ and *donor* (*MMP0108-MMP0109* with point mutagenesis of T$_{MMP0108}$ sequence)	This study
pMEV4-Cas9-M-T$_{rpl37\text{-}rpoP}$-sg	pMEV4- *Cas9* with sg targeting T$_{0249}$ and *donor* (*MMP0248-MMP0249* with point mutagenesis of T$_{MMP0249}$ sequence)	This study
pMEV4-Cas9-M-T$_{rpl21\text{-}rpoF}$-sg	pMEV4- *Cas9* with sg targeting T$_{0093}$ and *donor (MMP0092-MMP0093* with point mutagenesis of T$_{MMP0093}$ sequence*)*	This study

pMEV4-$P_{MMP0386}$-mCherry-pac-$T_{MMP1559}$	pMEV4 containing promoter of *MMP0386* , *mCherry*, Pur^R, terminator of *MMP1559*	This study
pMEV4-$P_{MMP0386}$-*rpoP*-*pac*-$T_{MMP1559}$	pMEV4 containing promoter of *MMP0386* , *MMP0248* ORF, Pur^R, terminator of *MMP1559*	This study
pMEV4-$P_{MMP0386}$-*rpoF*-*pac*-$T_{MMP1559}$	pMEV4 containing promoter of *MMP0386* , *MMP0092* ORF, Pur^R, terminator of *MMP1559*	This study
pET28a	Kana^R	This study
pET28a-*rpl37Ae*	pET28a with *MMP0248* inserted, Kana^R	This study
pET28a-*rpl21e*	pET28a with *MMP0093* inserted, Kana^R	This study
pET28a-*rpoP*	pET28a with *MMP0249* inserted, Kana^R	This study
pET28a-*afuA*	pET28a with *MMP0108* inserted, Kana^R	This study
pET28a-*rpoA''*	pET28a with *MMP1329* inserted, Kana^R	This study
pGEX-4T-1	Amp^R	Takara, Japan
pGEX-4T-1-*rpoF*	pGEX-4T-1 with *MMP0092* inserted , Amp^R	This study
pGEX-4T-1-*afuC*	pGEX-4T-1 with *MMP0110* inserted, Amp^R	This study

Table S2. Primers used in this study

Primers	Sequence（5'-3'）	Purpose
pET28a-F	CTCGAGCACCACCACCAC	Recombinant protein purification
pET28a-R	GGTATATCTCCTTCTTAAAGTTAAACAAAATTATTTCTAGAGGGGAATTGTT	Recombinant protein purification
afuA-pET28a-F	CTTTAAGAAGGAGATATACCTTGAAAAATTTAAAAGGCATACTC	Construction of plasmid pET28a-*afuA*
afuA-pET28a-R	CAGTGGTGGTGGTGGTGGTGCTCGAGTTCTGTAGTTGAAATTTCTCTATATC	Construction of plasmid pET28a-*afuA*
rpL37Ae-pET28a-F	CTTTAAGAAGGAGATATACCATGGTAGAATTTAGCCACAC	Construction of plasmid pET28a- *rpl37*
rpL37Ae-pET28a-R	AGTGGTGGTGGTGGTGGTGCTCGAGGATTTCTCTACTTTTACTTTCGA	Construction of plasmid pET28a-*rpl37*
rpL21e-pET28a-F	CTTTAAGAAGGAGATATACCATGCAAAAAAGTGAAGGATTTAG	Construction of plasmid pET28a-*rpl21*
rpL21e-pET28a-R	AGTGGTGGTGGTGGTGGTGCTCGAGTAATTTAGATTCTCTTAAGTGTTGC	Construction of plasmid pET28a-*rpl21*
rpoP-pET28a-F	CTTTAAGAAGGAGATATACCATGGCAGAGTACAAATGTTCCA	Construction of plasmid pET28a-*rpoP*
rpoP-pET28a-R	AGTGGTGGTGGTGGTGGTGCTCGAGCTTGCCTGAACTTTTTTAACTAT	Construction of plasmid pET28a-*rpoP*
rpoA2-pET28a-F	CTTTAAGAAGGAGATATACCATGCAAATGGCCGATTTAGAAAA	Construction of plasmid pET28a-*rpoP*
rpo A2-pET28a-R	AGTGGTGGTGGTGGTGGTGCTCGAGTAATTCTTTTCCTTCTTCGTAACTCTT	Construction of plasmid pET28a-*rpoP*
EcoRI-*afuC*-F	CCGGAATTCATGGATTTAAGACTCGAAAACATTTCA	Construction of plasmid pGEX-4T-1-*afuC*
XhoI-*afuC*-R	CCGCTCGAGTTAAATTGAAATTATACTGTTTTTATCAAATTCAAACA	Construction of plasmid pGEX-4T-1-*afuC*
EcoRI-*rpoF*-F	CCGGAATTCATGATTGGAAAAGAAATTATTTCCG	Construction of plasmid pGEX-4T-1-*rpoF*
XhoI- *rpoF*-R	CCGCTCGAGTTATTTGAATTTACTAACTACTTCTAAAAT	Construction of plasmid pGEX-4T-1-*rpoF*
rplL-donor-F	TTGAAGCAGTTGCAGCTATG	Construction of stain *E. coli* M-$T_{rplL-rpoBC}$
rplL-donor-R	TACTGCCCTTCAGGATCTTGC	Construction of stain *E. coli* M-$T_{rplL-rpoBC}$
rplL-Mu-donor-F	GGCTGGTGACTTTTTGGCTGGTGACTTTTTTGCGCTGTAAGGCGCC	Construction of stain *E. coli* M-$T_{rplL-rpoBC}$
rplL-Mu-donor-R	ACAGCGCAAAAAAGTCACCAGCCAAAAAGTCACCAGCCATCAGCCTGAT	Construction of stain *E. coli* M-$T_{rplL-rpoBC}$
rplL-sg-pEcgRNA-	AGGTATAATACTAGTACCAGCCTTTTTGCGCTGTA	Construction of stain *E. coli*

F	GTTTTAGAGCTAGAAATAGCAAG	M-$T_{rplL-rpoBC}$
rplL-sg-pEcgRNA-R	TCTAGCTCTAAAACTACAGCGCAAAAGGCTGGTACTAGTATTATACCTAGGACTGAGC	Construction of stain *E. coli* M-$T_{rplL-rpoBC}$
pMEV4-TArm-F	GAGAATAGAAAGTACATTATATTGC	Construction of ioTTS terminator mutagenesis strains
pMEV4-TArm-R	TCCTGTTCCAAGGATTCCAAC	Construction of ioTTS terminator mutagenesis strains
T0093Arm-pMEV4-F	TTGGAATCCTTGGAACAGGAGCAGAAAATATCTAAAATAGAAATGGT	Construction of stain *MMP M-$T_{rpl21-rpoF}$*
T0093Arm-pMEV4-R	ATAATGTACTTTCTATTCTCCTAATTGGTTTAATAATAAAATGGTAGA	Construction of stain *MMP M-$T_{rpl21-rpoF}$*
SgT0093-pMEV4-F	ATTATACTTGTTTAGACTAATCTGTTTTTGGTTTTAGAGCTAGAAATAGCAAG	Construction of stain *MMP M-$T_{rpl21-rpoF}$*
SgT0093-pMEV4-R	GCTCTAAAACTTTAGACTAATCTGTTTTTGCAAGTATAATTACTAATCAGCAATATAATG	Construction of stain *MMP M-$T_{rpl21-rpoF}$*
T0093Mu-donor-F	TAAATTATAACGAATCCAGATATCTAGACGACAGACTAATCTGTTTTTGAGGGAGAT	Construction of stain *MMP M-$T_{rpl21-rpoF}$*
T0093Mu-donor-R	AGATTAGTCTGTCGTCTAGATATCTGGATTCGTTATAATTTAAGATTCTCTTAAGTGTT	Construction of stain *MMP M-$T_{rpl21-rpoF}$*
T0249Arm-pMEV4-F	TCCAAGGTTTTTAAGTTATTTACGGATAGAAACAGGTG	Construction of stain *MMP M-$T_{rpl37-rpoP}$*
T0249Arm-pMEV4-R	ATAATGTACTTTCTATTCTCAAACTTTGAACCCAGTTTTC	Construction of stain *MMP M-$T_{rpl37-rpoP}$*
T0249Mu-F	TTTTAAACCAUACAUAACUCAUAAGAAGCUAACAUAGGTGATATTATGGCAGAG	Construction of stain *MMP M-$T_{rpl37-rpoP}$*
T0249Mu-R	AATATCACCTATGTTAGCTTCTTATGAGTTATGTATGGTTTAAAAATAATTAAATTAGATTTCTC	Construction of stain *MMP M-$T_{rpl37-rpoP}$*
SgT0249-pMEV4-F	ATTATACTTGAGCTTAAAATGAAAAATAAAGTTTTAGAGCTAGAAATAGCAAG	Construction of stain *MMP M-$T_{rpl37-rpoP}$*
SgT0249-pMEV4-R	GCTCTAAAACTTTATTTTTCATTTTAAGCTCAAGTATAATTACTAATCAGCAATATAAT	Construction of stain *MMP M-$T_{rpl37-rpoP}$*
afuA-donor-pMEV4-F	TCCAAGGTTTTTAAGTTATCCTGGAAATTTGGTAACTGC	Construction of stain *MMP M-T_{afuABC}*
afuA-donor-pMEV4-R	ATAATGTACTTTCTATTCTCGTGCCTTCCAAACAGGAAAAC	Construction of stain *MMP M-T_{afuABC}*
Sg-afua-pMEV4-F	ATTATACTTGTTTTTTACTTATTTTTTTTCGTTTTAGAGCTAGAAATAGCAAG	Construction of stain *MMP M-T_{afuABC}*
Sg-afua-pMEV4-R	GCTCTAAAACGAAAAAAAATAAGTAAAAAACAAGTATAATTACTAATCAGCAATATAATG	Construction of stain *MMP M-T_{afuABC}*
Tafua-Mu-F	CAACTACAGAATAAACAGGACACTTACGACACCGCAGGTGCAGTAAATGAAAGAAGT	Construction of stain *MMP M-T_{afuABC}*
Tafua-Mu-R	TTACTGCACCTGCGGTGTCGTAAGTGTCCTGTTTA	Construction of stain *MMP*

	TTCTGTAGTTGAAATTTCTCTATA	$M\text{-}T_{afuABC}$
pMEV4-0386-F	ACTAGTAGCGGCCGCTGC	Construction of overexpression strains
pMEV4-0386-R	AGCGCTACCTGCACTCTAG	Construction of overexpression strains
rpoP-p0386-F	CTAGAGTGCAGGTAGCGCTATGGCAGAGTACAAATGTTC	Construction of *rpoP* overexpression strain
rpoP-p0386-R	CTGCAGCGGCCGCTACTAGTTTATCTTGCCTGAACTTTTTTAAC	Construction of *rpoP* overexpression strain
rpoF-p0386-F	ACTAGAGTGCAGGTAGCGCTATGATTGGAAAAGAAATTATTTCCGA	Construction of *rpoF* overexpression strain
rpoF-p0386-R	CTGCAGCGGCCGCTACTAGTTTATTTGAATTTACTAACTACTTCTAAAA	Construction of *rpoP* overexpression strain
mCherry-P0386-F	ACTAGAGTGCAGGTAGCGCTATGGTTTCAAAAGGAGAAGAAG	Construction of *mCherry* expression strain
mCherry-P0386-R	CTGCAGCGGCCGCTACTAGTTTATTTGTATAATTCATC	Construction of *mCherry* expression strain

Table S3. Primers used in quantitative RT-PCR

Mmp-16S rRNA-qF	CGATCGGTACGGGCCTTGAGAGAG
Mmp-16S rRNA-qR	CACCGAACTTGCCCAGCCCTTATT
MMP0290-qF	CGGAATGGATGCCGAAGAT
MMP0290-qR	GGTTTGAAACCATGTCGATGTC
MMP0291-qF	CCCAGAATCCGGAATTACAATGG
MMP0291-qR	CTGCAACTACAACGTGTTCTG
MMP1635-qF	TGAAGTATTTACATCACCAATGTGT
MMP1635-qR	AGCTCCAACAAATTTAACTTCCC
MMP1634-qF	TGTTGTTGGTGCAAGCTGT
MMP1634-qR	TCTTGTAATTTCCATCATCCGTCT
MMP0093-qF	GAATGGCTCCTTTAACAAGAGCT
MMP0093-qR	TCTTTGTATTTTCCGCCATCT
MMP0092-qF	GGATGAAAGAGCAGATTTTGACGA
MMP0092-qF	GGATGAAAGAGCAGATTTTGACGA
MMP0094-qF	CCCGACATTGTGGTTATGGTGA
MMP0094-qR	TCTTCAACCGAGGTCATGTACT
MMP1189-qF	AGGCTGCAAAACTTGTAAAAGATG
MMP1189-qR	CGGAATTCCAGAATTACTTGCCAT
MMP-atpK-qF	CAGAAACTCAGGCAATTTACGGT
MMP-atpK-qR	CCCATTGCATCAGGATCTCTAGC
MMP-atpE-qF	AGCTGAAGCAAGACTCTCCG
MMP-atpE-qR	AACAATTCTAATTCGCCGCCG
MMP-atpF-qF	CAGACTTGCTGGACTTACAGATGT
MMP-atpF-qR	GGACCGTTTTTGTCAGGAACT
MMP-atpA-qF	CCCAGATGTTCGAAGTGGTTAAA
MMP-atpA-qR	TGCTTTTAACATACCAGGGCCG
MMP-afuA-qF	TGGCCATCCCCAGTTTCGAT
MMP-afuA-qR	TCCATGTCGAGGAAATCAAGATC
MMP-afuB-qF	ACGGTTTTACGCTTGATGCA
MMP-afuB-qR	CCCGGGGGTAATTATTGGTAG
eco-16S-qF	GGGAGGAAGGGAGTAAAGTT
eco-16S-qR	CAGTTCCCAGGTTGAGCCCG
rplL-eco-qF	TCGGTGTTTCCGCTGCTGCT
rplL-eco-qR	GCCGGTGCAGATTCTACCAG
rpoB-eco-qF	GAGCAAGATCCTGAAGGGCA
rpoB-eco-qR	GCAGTTTAACGCGCAGCGG
MMP0250-5'-qF	GAAGGACAGCCAATAGGCAT
MMP0250-5'-qR	CTTTTCGCTGGTTAGCTGTTAATTG
rpl37Ae-3'-qF	CAGCAGGAAGATTTGGATCAAGAT
rpl37Ae-3'-qR	GGAGTGTATGCTCCACCAGC
rpoP-5'-qF	ATGGCAGAGTACAAATGTTCCAAC

rpoP-5'-qR	CAAGGCTTCTGGTTCTTTGAGAAG
MMP0246-3'-qF	GGCTCAGAAAATATGGGTCCG
MMP0246-3'-qR	GGTCCATTTTGCACTTTGATCG
MMP0245-5'-qF	ATGGAATTACCTGCAAACGTTC
MMP0245-5'-qR	CCACCAGCCATCTTAAATACTTCT

Table S4. Probes used in the northern blot assays

MMP0290-mid-probe	GCTTTTCCAGTAATCGAATAAGTTTATTTCCAAGCATGTCCATTACCTGAACTTTTGG
MMP0291-5'-probe	CCACTGCTTCGACAAATCTTCTTGCCCATCCTTCATCAGTGTCTTTAACTTCAATCACT
MMP0094-3'-probe	CGGGCGTTCTTTGATCAATCGTCAAATCTTCGAGTTTTTTACTAATTCGGCTACTTCT
MMP0093-3'-probe	TCCGCCATCTTTAACTCTAACTAAGAATGAACTTCCTCTTTGAGCAACAACAACTCCAG
MMP0092-mid-probe	CAGTTAAACCTAAATTTACGAGCTGTTCAAACATAGCGTCAGCATCGTCTTTACTTATT
MMP1635-mid-probe	CAACGGTAGGAACTGCCATAATTCCAAGTTCAGCAGCCTTTTCAGGGTGTTCCATTAC
MMP1634-5'-probe	TGGGAATCCCCATCGGTTTCGATCAACAAAATTCCGCCTTTCGTTTTCTCAACTACTTT
MMP1190-3'-probe	TTACCGCAGGTTCTTCACCAGCAACAATTACCATTCCTTCTTCAGGTTCAAAGTCTGC
MMP1189-5'-probe	TTCCAAGTTCCTGAATGAAAAGATTTGCAGTTGAACCCGAACCAAGTCCCACAACCAT
MMP0250-3'-probe	CAGCGGCCAAGATTTCTGAGATGCCTATTGGCTGTCCTTCTTTAAATTTAGCTGCCAAG
rpoP-5'-probe	GCTTTTAATCCTATTTCATCAAGAGTTACGATTTTTCCGCAGTTGGAACATTTGTACTC
rpl37Ae-3'-probe	GATTTCTCTACTTTTACTTTCGATAACTCTTCTGATTGCTTTTGTAACAACTTTACCAG
MMP0245-5'-probe	TGCTGCTGGAGTTGTTGGAATTGCATTAATTGATTTTGAACGTTTGCAGGTAATTCCAT
MMP0251-5'-probe	CAGCTTCCTCTTCTTCAGCCTTAACTTTTTCGTAAACTGCTTTTACGATTTTTTCAATT

Supplementary References

1 Li, Q. *et al.* A modified pCas/pTargetF system for CRISPR-Cas9-assisted genome editing in *Escherichia coli*. *Acta Biochim Biophys Sin (Shanghai)* **53**, 620-627, doi:10.1093/abbs/gmab036 (2021).

2 Sarmiento, F., Leigh, J. A. & Whitman, W. B. Genetic systems for hydrogenotrophic methanogens. *Methods Enzymol* **494**, 43-73, doi:10.1016/B978-0-12-385112-3.00003-2 (2011).

3 Li, J. *et al.* CRISPR-Cas9 Toolkit for genome editing in an autotrophic CO_2-fixing methanogenic archaeon. *Microbiology Spectrum*, doi:ARTN e01165-2210.1128/spectrum.01165-22 (2022).
